# Disease-associated nonsense and frame-shift variants resulting in the truncation of the GluN2A or GluN2B C-terminal domain decrease NMDAR surface expression and reduce potentiating effects of neurosteroids

**DOI:** 10.1007/s00018-023-05062-6

**Published:** 2024-01-12

**Authors:** Bohdan Kysilov, Viktor Kuchtiak, Barbora Hrcka Krausova, Ales Balik, Miloslav Korinek, Klevinda Fili, Mark Dobrovolski, Vera Abramova, Hana Chodounska, Eva Kudova, Paulina Bozikova, Jiri Cerny, Tereza Smejkalova, Ladislav Vyklicky

**Affiliations:** 1 Laboratory of Cellular Neurophysiology, Institute of Physiology of the Czech Academy of Sciences, Videnska 1083, 14200 Prague 4, Czech Republic; 2 Institute of Organic Chemistry and Biochemistry of the Czech Academy of Sciences, Flemingovo Nam. 2, 16610 Prague 6, Czech Republic; 3 Institute of Biotechnology of the Czech Academy of Sciences, BIOCEV, Prumyslova 595, 25250 Vestec, Czech Republic; 4 Faculty of Science, Charles University, Albertov 2038, 12800 Prague 2, Czech Republic; 5 Third Faculty of Medicine, Charles University, Ruska 87, 10000 Prague 10, Czech Republic; 6 Present Address: Stony Brook University, Stony Brook, 100 Nicolls Road, NY 11794 USA

**Keywords:** Glutamate receptors, Channelopathy, Endogenous neuroactive steroid, *GRIN2* genes, Surface expression, Rescue pharmacology

## Abstract

*N*-methyl-d-aspartate receptors (NMDARs) play a critical role in normal brain function, and variants in genes encoding NMDAR subunits have been described in individuals with various neuropsychiatric disorders. We have used whole-cell patch-clamp electrophysiology, fluorescence microscopy and in-silico modeling to explore the functional consequences of disease-associated nonsense and frame-shift variants resulting in the truncation of GluN2A or GluN2B C-terminal domain (CTD). This study characterizes variant NMDARs and shows their reduced surface expression and synaptic localization, altered agonist affinity, increased desensitization, and reduced probability of channel opening. We also show that naturally occurring and synthetic steroids pregnenolone sulfate and epipregnanolone butanoic acid, respectively, enhance NMDAR function in a way that is dependent on the length of the truncated CTD and, further, is steroid-specific, GluN2A/B subunit-specific, and GluN1 splice variant-specific. Adding to the previously described effects of disease-associated NMDAR variants on the receptor biogenesis and function, our results improve the understanding of the molecular consequences of NMDAR CTD truncations and provide an opportunity for the development of new therapeutic neurosteroid-based ligands.

**Supplementary Information:**

The online version contains supplementary material available at 10.1007/s00018-023-05062-6.

## Introduction

*N*-methyl-d-aspartate-receptors (NMDARs) are ionotropic glutamate receptors that mediate a slow component of excitatory synaptic transmission in virtually all central nervous system circuits to regulate physiological functions, including synaptic plasticity [1]. NMDARs are heterotetramers, composed of four subunits—two GluN1 (existing in eight alternatively spliced isoforms) and two GluN2(A-D) and/or GluN3(A-B) subunits. NMDARs have a modular design that includes two extracellular domains (amino-terminal domain (ATD) and agonist-binding domain (ABD)), a transmembrane domain (TMD), and a cytoplasmic C-terminal domain (CTD) [2]. GluN1 CTD is relatively short and, depending on the splice variant, it comprises 6–12% of the subunit mass, in contrast to GluN2A/B CTD, which comprises 44% of the subunit mass. Structural analysis indicates that NMDAR CTDs will be mostly intrinsically disordered [3, 4]. The CTDs are subject to numerous post-translational modifications and form protein–protein and protein–lipid interactions that link the cytoplasmic part of the NMDAR to a large network of postsynaptic signaling and scaffolding proteins [4]. The importance of the GluN2A/B CTD has been shown in knock-in mice expressing GluN2A subunits with truncated CTDs that survive into adulthood but have synaptic plasticity deficits, and mice expressing truncated GluN2B CTDs that die perinatally [5–7].

NMDARs lacking GluN2 CTDs are functional; however, detailed electrophysiological analysis revealed increased macroscopic desensitization, reduced peak open probability, and faster deactivation compared to wild type (WT) NMDARs [8, 9]. The mechanism by which the CTD affects the gating kinetics of the NMDAR is not well understood since swapping the CTDs of GluN2A and GluN2B subunits did not change the macroscopic responses. Our recent study indicates that the probability of opening and the gating kinetics of GluN1/GluN2B receptors are controlled by palmitoylation-mediated anchoring of the juxtamembrane portion of the GluN2B CTD to the membrane [10].

Given the broad physiological importance of NMDARs, it is unsurprising that variants in genes encoding NMDAR subunits (*GRIN1-3*) are associated with diverse brain disorders [11–13]. These variants occur in all receptor domains and are differently tolerated depending on the location [14]. Variants affecting the CTD have been found in individuals diagnosed with schizophrenia, epilepsy, aphasia, autism spectrum disorder, intellectual disability, and developmental delay [15].

To understand the consequences of *GRIN* gene variants on neuronal activity, we have studied a range of nonsense and frame-shift (indel) variants in the DNA sequence encoding the CTD of GluN2A and GluN2B subunits. We show that disease-associated variants alter NMDAR surface expression, synaptic localization, receptor affinity for agonists glutamate and glycine, desensitization, and deactivation. We used site-directed mutagenesis in the CTD to reveal that the effects of endogenous and synthetic neurosteroids with a potentiating effect at NMDARs, pregnenolone sulfate (PE-S) and epipregnanolone butyrate (EPA-But), differ depending on the site of the CTD truncation, are dependent on the splice variant of GluN1 and the palmitoylation state of the GluN2B CTD. This study of the disease-associated variants provides broader insights into the molecular mechanisms by which the CTD modifies NMDAR function and pharmacology and regulates receptor cellular location and abundance.

## Materials and methods

### DNA constructs

We have used the following genes encoding NMDA receptor subunits: rat GluN1-1a (rGluN1-1a, GeneBank accession number U08261), rGluN1-2a (U08262), rGluN1-3a (U08265), rGluN1-4a (U08267), rGluN2A (D13211), and rGluN2B (M91562) (in the expression vector pcDNA3.1); human GluN1-1a subunit (hGluN1˗1a; NM_007327), hGluN2A (NM_001134407), and hGluN2B (NM_000834) (in the expression vector pCI-neo; generous gift from prof. Traynelis). In addition, we used the expression vector pQBI 25 containing the gene for soluble eGFP (Takara, Tokyo, Japan).

For fluorescence microscopy, the eGFP tag was introduced into the human GluN2A and GluN2B constructs in the extracellular portion of the receptor after the first 24 amino acids (the signal peptide), with a peptide linker Ala-Ala-Val-Ala-Thr (GCCGCGGTCGCCACC) on the N-terminus and a Ser on the C-terminus of eGFP, by In Vivo Assembly cloning [16]. Subsequently, human versions of the GluN2A and GluN2B subunits with the eGFP tag (eGFP-hGluN2A/B subunits) were further transferred by restriction enzyme cloning into a pLEX-MCS expression vector (OpenBiosystem, Thermo Scientific) for lentiviral particle production and subsequent expression of eGFP-hGluN2A/B subunits in neurons.

Variants were introduced into GluN constructs (pCI-neo; pcDNA3.1; eGFPtag-pCI-neo; eGFPtag-pLEX-MCS) by site-directed mutagenesis or by In Vivo Assembly mutagenesis [16]. Site-directed mutagenesis was performed using the QuikChange II XL Site-Directed Mutagenesis Kit (Agilent Technologies, Santa Clara, CA, USA) in accordance with the manufacturer’s instructions. Primers were designed manually a purchased from Eurofins Genomics (Germany). After PCR, DpnI-treated product was transformed into ultracompetent XL10-Gold *E. coli* cells; overnight cultures were grown from several clones, and isolated DNA plasmids were sequenced to verify the presence of the variant (Eurofins Genomics).

Amino acids are numbered according to the full-length protein, including the signal peptide, with the initiating methionine as number 1.

### Transfection and maintenance of cells

Human embryonic kidney 293 (HEK) and monkey COS-7 cells were cultured in Dulbecco’s Modified Eagle Medium supplemented by Fetal Bovine Serum (10%; Merck) and Pen/Strep (Merck). Surface expression experiments were performed 24 h after the transfection of HEK cells or 48 h after the transfection of COS-7 cells with cDNA encoding hGluN1-1a and eGFP-hGluN2A or eGFP-hGluN2B subunits using GenJet transfection reagent (SignaGen) according to the manufacturer’s protocol. Electrophysiology experiments were performed 24–48 h after the transfection of HEK cells with cDNA encoding either rat or human versions of GluN1-1a, GluN1-2a, GluN1-3a, GluN1-4a, GluN2A or GluN2B subunits, and eGFP using Matra-A reagent (IBA, Göttingen, Germany) as described previously [17].

Primary neuronal cultures were prepared from mouse hippocampus on the first postnatal day by triturating cells after partial trypsin digestion. Neurons were cultured (37 500 cells/cm^2^) on poly-l-lysine-coated coverslips (0.1 mg/ml) in Minimum Essential Medium (Gibco™) supplemented by D-Glucose (1%), Pen/Strep (Merck), Sodium Pyruvate (Gibco™), N-2 Supplement (Gibco™), HEPES (20 mM), and Horse Serum (10%; Gibco™) for the first 24 h (1 day in vitro; DIV 1). The next day, all medium was replaced with Neurobasal medium (Gibco™) supplemented by l-Glutamine (0.5 mM), B27 supplement (Gibco™), β-Mercaptoethanol (25 μM), and basic fibroblast growth factor (10 ng/ml; Alomone). Half of the Neurobasal medium was replaced on DIV 7.

Neurons were transduced by lentiviruses containing genes for the expression of eGFP-hGluN2A or eGFP-hGluN2B subunit. Lentiviral particles were produced in transfected HEK cells using the GenJet transfection reagent with the following constructs: pLEX-MCS containing the gene encoding the eGFP-hGluN subunit, packaging plasmid (pCMVΔ8.1), and envelope plasmid (pMD.VSVG) at a ratio of 3.5:2.5:1. 24 h after transfection, media were collected for the first time, stored at 4 °C and replaced by fresh media. 72 h after transfection media were collected for the second time. The collected media were filtered through a cellulose acetate membrane filter (0.45 μm pore size). Lentiviruses were concentrated by ultracentrifugation of media at 50,000*g* for 2.5 h.

### Analysis of surface expression by fluorescence microscopy

The analysis of surface expression of NMDARs was performed in HEK cells. eGFP-hGluN2 subunit constructs were co-transfected with the hGluN1-1a subunit. The culture medium contained NMDAR antagonists AP-5 (50 μM), Mg^2+^ (20 mM), and ketamine (1 μg/ml). One day after transfection, the cells were washed with PBS, fixed with 4% paraformaldehyde, and blocked for 30 min (PBS containing 50 mM NH_4_Cl, 10% goat serum, and 2% fish gelatin). To evaluate NMDAR surface expression, cells were initially incubated with a primary rabbit anti-GFP antibody (1:1000; AB3080P; Merck), then washed with PBS and further incubated with a secondary goat anti-rabbit antibody conjugated with Alexa Fluor 647 (1:1000; A-21244; Invitrogen). To assess total intracellular receptor expression, cells were further washed with PBS and permeabilized using a permeabilization-blocking solution additionally containing 0.1% TritonX-100 and 0.1% Tween20 for 30 min. Intracellular receptors were stained using the same primary rabbit anti-GFP antibody followed by a secondary goat anti-rabbit antibody conjugated with Alexa fluor 555 (1:1000; A-21428; Invitrogen). To visualize the cell nucleus, 4′,6-diamidino-2-phenylindole (DAPI) fluorescent dye (1:2000; D1306; Invitrogen) was used. Cells were mounted with ProLong-Glass mounting medium. The images were acquired on a Leica DMi8 S microscope equipped with sCMOS camera (Leica DFC9000 GTC) and captured as z-stack images (z-step size: 0.18 µm) with PlanAPO 63x (1.47 NA) oil-immersion objective (Leica) at 395 nm (DAPI), 555 nm (intracellular staining), and 647 nm (surface staining) excitation wavelengths. Dead cells were excluded based on the DAPI staining of the nucleus. The images were analyzed using ImageJ software as the maximum projection of the z-stacks, where the average intensity of signals in channels 555 and 647 was measured (the intensity of the background was subtracted). Surface expression of NMDARs was determined as the ratio of these intensities, i.e., surface-to-intracellular signals.

### Quantitative (colorimetric) assay of surface expression

A quantitative surface expression assay was performed using COS-7 cell cultures co-transfected with hGluN1-1a and eGFP-hGluN2 subunits. The experiment was performed on two 24-well plates, which provided quadruplicates (divided into two duplicates per plate) for determining surface or total receptor expression. In addition, to determine the background signal, cells were transfected only with the construct containing hGluN1-1a subunit. Half of each plate was incubated with the surface antigen blocking solution (PBS containing 50 mM NH_4_Cl, 10% goat serum, 2% fish gelatine) and the other half with the permeabilizing blocking solution (PBS containing 50 mM NH_4_Cl, 10% goat serum, 2% fish gelatine, 0.1% TritonX-100, 0.1% Tween20). Subsequently, the cells were incubated with a primary rabbit anti-GFP antibody (1:500; 11-476-C100; Exbio, Czech rep.) followed by a secondary goat anti-rabbit horseradish peroxidase-conjugated antibody (1:500; AQ132P; Sigma-Aldrich). Afterwards, o-phenylenediamine dihydrochloride solution (SIGMAFAST OPD tablet set; P9187; Sigma-Aldrich) was added, and cells were incubated in the dark for 30 min. The absorbance was measured at 492 nm using an Infinite PRO 200 multifunction modular reader (Tecan). Values of surface and total expression were obtained after subtracting the background (hGluN1-1a subunit expressed alone). To obtain the value of intracellular expression, the values of surface expression were subtracted from the total expression values. Surface expression was determined as the ratio of surface-to-intracellular values.

### Analysis of surface expression and synaptic localization in neurons

At DIV 4, lentiviral particles containing an eGFP-hGluN2 subunit-expressing construct were administered to the primary neuronal culture. At DIV 14, surface and intracellular expression of the eGFP-hGluN2A/B subunits were determined in neurons by the protocol described for HEK cell cultures with the following exceptions. All antibodies were diluted 1:500. Secondary goat anti-rabbit Alexa Fluor 488-conjugated antibody (A-11034; Invitrogen) was used for the staining of intracellular hGluN2A/B. In addition, synapses were labeled with a primary mouse anti-PSD-95 antibody (1:500; K28/43; NeuroMab) followed by a secondary goat anti-mouse Alexa Fluor 555-conjugated antibody (1:500; A-21422; Invitrogen). Similarly to HEK cells, the images from neurons were acquired with a Leica DMi8 S microscope equipped with a sCMOS camera (Leica DFC9000 GTC) as z-stack images (z-step size: 0.18 um) with PlanAPO 63x (1.47 NA) oil-immersion objective (Leica) at 395 nm (DAPI), 488 nm (eGFP-hGluN2 intracellular staining), 555 nm (PSD-95), and 647 nm (eGFP-hGluN2 surface staining) excitation wavelengths. An additional experiment verified that the native fluorescence intensity of the eGFP protein is negligible compared to the intensity of Alexa Fluor 488. The obtained images were then deconvolved by Huygens software using the Classic Maximum Likelihood Estimation algorithm and further analyzed using ImageJ software: (1) Surface receptor expression was analyzed from the average intensities of signals in channels 488 and 647 (after subtracting the intensity of the background) in the somata of neurons. Surface expression was determined as the ratio of these intensities, i.e., surface-to-intracellular signals. (2) The percentage of surface receptors overlapping with PSD-95 (indicating synaptic localization) was calculated from 20 µm long region of secondary dendrites. The analysis was done automatically by our macro. After the maximum projection, adaptive thresholding (ImageJ plugin) was used to overcome the limitation of the conventional threshold method when feature intensities were not homogeneous. After the creation of masks of surface receptors and PSD-95, the percentage of overlapping pixels was calculated. In addition, our macro-incorporated plugin “Analyze Particles” automatically counts the number of puncta of surface receptor and PSD-95 clusters.

### Electrophysiology

Whole-cell responses were recorded using a patch-clamp amplifier (Axopatch 200B; Molecular Devices) after series resistance (< 10 MΩ) and capacitance compensation by 80–90%. All recordings were performed in the voltage-clamp mode at a holding potential of – 60 mV. A microprocessor-controlled multibarrel fast perfusion system was used for solution application, with a time constant of solution exchange around the cells of ~ 10 ms [18]. Agonist-induced responses were low-pass filtered at 2 kHz, digitally sampled at 5 kHz, and analyzed with pClamp software version 10.6 (Molecular Devices). Patch pipettes (3–5 MΩ) pulled from borosilicate glass were filled with a Cs^+^-based intracellular solution. Specifically, the intracellular solution for all experiments except the experiments involving a Ca^2+^ challenge contained (in mM): 120 gluconic acid delta lactone, 15 CsCl, 10 HEPES, 10 BAPTA, 1 CaCl_2_, 3 MgCl_2_, and 2 ATP-Mg salt, pH-adjusted to 7.2 with CsOH. For the Ca^2+^ challenge, the intracellular solution contained (in mM): 125 gluconic acid delta lactone, 15 CsCl, 10 HEPES, 5 EGTA, 0.5 CaCl_2_, 3 MgCl_2_, and 2 ATP-Mg salt (pH-adjusted to 7.2 with CsOH). Except for the experiments involving the Ca^2+^ challenge, the extracellular solution was as follows (in mM): 160 NaCl, 2.5 KCl, 10 HEPES, 10 glucose, 0.7 CaCl_2_, and 0.2 EDTA (pH-adjusted to 7.3 with NaOH). For the assessment of steroid modulatory effect before and after the Ca^2+^ challenge, the extracellular solution contained 0.3 CaCl_2_ and 0.1 EDTA. For the Ca^2+^ challenge, the extracellular solution contained 2 mM CaCl_2_ and 0.1 mM EDTA. NMDAR responses were induced by 1 μM, 3 μM, or 1 mM glutamate together with 30 μM glycine.

Steroids were dissolved in dimethyl sulfoxide and added to the extracellular solution at the indicated concentrations, with the final dimethyl sulfoxide concentration of 1%. An equivalent amount of dimethyl sulfoxide was present in control solutions. (5S,10R)-(+)-5-methyl-10,11-dihydro-5H-dibenzo[a,d]cyclohepten-5,10-imine hydrogen maleate **(**MK801) was dissolved in MilliQ water to obtain a stock solution of 2 mM and stored at − 20 °C. All drugs, unless otherwise stated, were purchased from Sigma-Aldrich. Experiments were performed at room temperature (21–25 °C).

### Data analysis

#### Analysis of the steroid effect

The degree of steroid modulation ($E$) was calculated as:

1 $$E = ((I_{\text{S}} - I_{\text{A}} )/I_{\text{A}} ) \times 100,$$

where $I_{\text{A}}$ is the amplitude of the agonist-induced response and $I_{\text{S}}$ is the amplitude of the response recorded in the presence of the agonist and the steroid. The degree of steroid modulation was determined in the presence of 1 μM glutamate for GluN2B-containing recombinant NMDARs or 3 μM glutamate for GluN2A-containing recombinant NMDARs.

#### Agonist dose–response analysis

Normalized amplitudes (*I*) measured in individual HEK cells were fit to the following logistic equation:

2 $$I = { }1/{ }(1 + ({\text{EC}}_{50} /\left[ {{\text{agonist}}} \right])^h ),$$

where ${\text{EC}}_{50}$ is the agonist concentration that produces a half-maximal response, $\left[ {{\text{agonist}}} \right]$ is the agonist concentration, and *h* is the Hill slope. The dose–response analysis for glutamate was determined in the presence of 30 μM glycine and the dose–response analysis for glycine was determined in the presence of 1 mM glutamate.

#### Analysis of desensitization

Desensitization $(D)$ of the WT or variant NMDARs was calculated as:

3 $$D = 100 \times \left( {1 - (I_{{\text{SS}}} /I_{\text{P}} )} \right),$$

where $I_{{\text{SS}}}$ is the steady state response and $I_P$ is the peak response to 1 mM glutamate.

#### Open probability

The channel open probability ($P_{\text{o}}$) was assessed from the kinetics of the MK­801 (1 µM) inhibition of responses to 1 mM glutamate that was fitted by the kinetic model (Eq. 4) using Gepasi software (version 3.21 [19–21]). The glutamate binding steps were not considered because in the presence of 1 mM glutamate, NMDAR exists with a high probability (> 99.6%) only in doubly liganded states with the channel closed (*C*) or open (*O*), and/or in the desensitized state (*D*). B represents the MK-801-blocked state of NMDAR.

4 $$D\underset{{k_{\text{d}} }}{\overset{{k_{\text{r}} }}{\rightleftharpoons}}C\underset{{k_{\text{c}} }}{\overset{{k_{\text{o}} }}{\rightleftharpoons}}O\underset{{k_{\text{u}} }}{\overset{{k_{\text{b}} }}{\rightleftharpoons}}B$$

The fitting procedure consisted of two steps [22]. In the first step, the response induced by 1 mM glutamate in HEK cells transfected with the WT or varinat NMDARs was analyzed for the peak ($I_{\text{P}}$) and steady state response ($I_{{\text{SS}}}$), and the onset of desensitization was determined by the single exponential function ($\tau_{\text{d}}$). Desensitization ($D$) and the kinetic constants describing the rate of desensitization ($k_{\text{d}}$) and resensitization ($k_{\text{r}}$) were determined from Eqs. 3, 5 and 6:

5 $$k_{\text{d}} = D/\tau_{\text{d}}$$

6 $$k_{\text{r}} = (1 - D)/\tau_{\text{d}}$$

In the second step, $k_{\text{d}}$ and $k_{\text{r}}$ were fixed at values obtained from the first step, and the closing rate ($k_{\text{c}}$) at an arbitrary value of 200 s^−1^. The NMDAR response recorded in the presence of 1 mM glutamate and 1 µM MK-801 was fitted to the model while the opening rate ($k_{\text{o}}$) was set as a free parameter. MK-801 blocking rate (*k*_b_) was set to 25 μM^−1^ s^−1^ [23–25] (Eq. 4). Macroscopic open probability (*P*_o_) was calculated as:

7 $$P_{\text{o}} = 100 \times k_{\text{o}} /(k_{\text{o}} + k_{\text{c}} )$$

### Steroid synthesis

The steroids were prepared by multistep synthetic procedures as described earlier: 20-oxo-pregn-5-en-3β-yl 3-sulfate (pregnenolone sulfate; PE-S) [26] and 4-(20-oxo-5β-pregnan-3β-yl) 3-butanoic acid (epipregnanolone butanoic acid; EPA-But) [27]; infrared, high-resolution mass spectrometry and nuclear magnetic resonance spectra confirmed the structures of all synthesized steroids.

### Homology modeling

The initial model of the human GluN1/GluN2B complex was prepared based on the previously created rat NMDAR homology model [28] originally derived from the available structures 4pe5, 4tll, and 4tlm [29, 30]. The receptor geometry corresponded to the ligand-activated state (RAA) as obtained from previous opening/closing simulations [31]. The model GluN1/GluN2B complex consisting of two full GluN1 subunits (Q05586 residues 23–938) and two GluN2B subunits with truncated CTD (Q13224 residues 30 to 877) was built using the *automodel* function of MODELLER 9.23 [32, 33] including the glycine and glutamate ligands in their corresponding ABDs.

A second model of GluN1/GluN2B complex was derived from the full model, introducing the GluN1 subunit in its GluN1-2a splice variant (Q05586-3 residues 23 to 901). This variant is missing the C1 cassette residues 864 to 900, numbered according to the canonical Q05586.

### Molecular dynamics (MD) simulations

The systems for the MD simulation of the human GluN1/GluN2B receptor model were prepared using CHARMM-GUI [34, 35]. The palmitoyl modifications of GluN2B cysteine residues C849, C854, and C871 were introduced and the receptor was inserted into a model membrane containing 1-palmitoyl-2-oleoyl-sn-glycero-3-phosphocholine and cholesterol (3:1). The simulation box (180 Å × 180 Å × 300 Å) was filled with TIP3P water and a charge-neutral system with a final salt concentration of 150 mM was created adding Na and Cl ions. The system was equilibrated using the default CHARMM-GUI equilibration protocol. MD simulations of 50, 150 ns, or 500 ns (2 fs time step, collecting geometry every 10 ps) were performed using GPU-accelerated Gromacs 2021.4 [36] and CHARMM36 forcefield [37] with the Nosé–Hoover thermostat (reference temperature 303.15 K, τ 1.0 with separate baths for the solute, membrane, and the solvent) and the Parrinello-Rahman barostat (reference pressure 1.0 atm, τ 5.0 and compressibility 4.5 × 10^–5^). The metadynamics simulations were carried out in Gromacs using the open-source, community-developed PLUMED library, version 2.7.3 [38, 39]. The bias (SIGMA = 0.20, HEIGHT = 0.20, PACE = 200) was added to the collective variable defined as the *z*-component (perpendicular to the membrane plane) of the distance between the center of mass of the C1 cassette residues and the center of mass of the TMD residues. Visualization of structures, graphical representation, and analysis of residues surrounding the selected regions were performed using the open-source version of PyMOL (Version 2.6.0a0).

### Statistical analysis

Data being compared (i.e., drug effect under control versus experimental conditions or for WT versus variant genotype) were collected on the same days in random order; *n* refers to the number of cells tested unless otherwise stated. Data obtained for WT receptors in different recording sessions were pooled, resulting in unequal sample sizes. Statistical analysis was performed with STATGRAPHICS Centurion 18 (Statgraphics Technologies, Inc., VA, USA). If the original data did not have symmetric distribution and constant variance, they were power transformed to attain symmetric distribution and homoscedasticity (constant variance). We used absolute values of studentized residuals greater than 3 to identify outliers. Data were further analyzed by the Analysis of variance (ANOVA) followed by multiple comparisons versus control (WT) using Dunnett’s method or by pairwise comparisons using Duncan method. *p* ≤ 0.05 was considered statistically significant throughout the study. Data are presented as mean ± standard error of the mean (SEM).

## Results

### Rare variants in the CTD affect NMDAR surface expression

Nonsense and frame-shift variants in the DNA encoding NMDAR subunits result in a premature stop codon and a truncated protein. Such variants were identified in *GRIN2A* and *GRIN2B* genes in individuals diagnosed with neurodevelopmental disorders. We screened the available databases (https://www.ncbi.nlm.nih.gov/clinvar and http://functionalvariants.emory.edu/database/index.html)) and selected 10 de-novo nonsense and frame-shift variants (Table 1) to investigate the consequences of subunit truncation for the receptor surface expression, synaptic localization, function, and pharmacology. In addition, we have also included artificial hGluN2A(R846X) variant, which has not been found in humans but allows the analysis of receptors with the entire hGluN2A CTD deleted, comparably to hGluN2B(R847X) variant.

**Table 1:** Selected de-novo nonsense and frame-shift variants in the hGluN2A and hGluN2B CTDs and their phenotypic characteristics

Subunit	Variant	Type	Phenotype	References
hGluN2A	S913X	Nonsense	EPI, ID	[40]
	Y943X	Nonsense	EPI, ID	[41]
	Q950X	Nonsense	ANS	[42]
	Y1387X	Nonsense	EPI, ID, ASD	[43]
hGluN2B	R847X	Nonsense	ASD, ID, DD	[44, 45]
	I864SfsX20	Frame-shift	ANS, DD, ID	[42]
	Y1004X	Nonsense	ASD, ID	[45]
	R1099AfsX51	Frame-shift	ASD, ID	[46]
	Y1155X	Nonsense	ASD, ID	[47, 48]
	S1415X	Nonsense	ASD	(DB)

X, stop codon; fs, frame-shift; ANS, abnormality of the nervous system; ASD, autism spectrum disorder or features thereof; DD, developmental delay; EPI, epilepsy, focal or generalized seizures; ID, intellectual disability (includes non-verbal); DB, Functional Variants CFERV Database (http://functionalvariants.emory.edu/database/index.html)

We used immunofluorescence microscopy and a colorimetric assay to analyze the effects of the selected nonsense and frame-shift variants on the surface expression of variant NMDAR subunits. First, we generated constructs encoding eGFP-tagged hGluN2A/B subunits (see Methods). In the second step, the eGFP-tagged subunits (WT or variant) were co-transfected with the hGluN1-1a (hGluN1) subunit into HEK cells (immunofluorescence microscopy) or COS-7 cells (colorimetric assay) and NMDAR surface expression was analyzed.

**Fig. 1:**
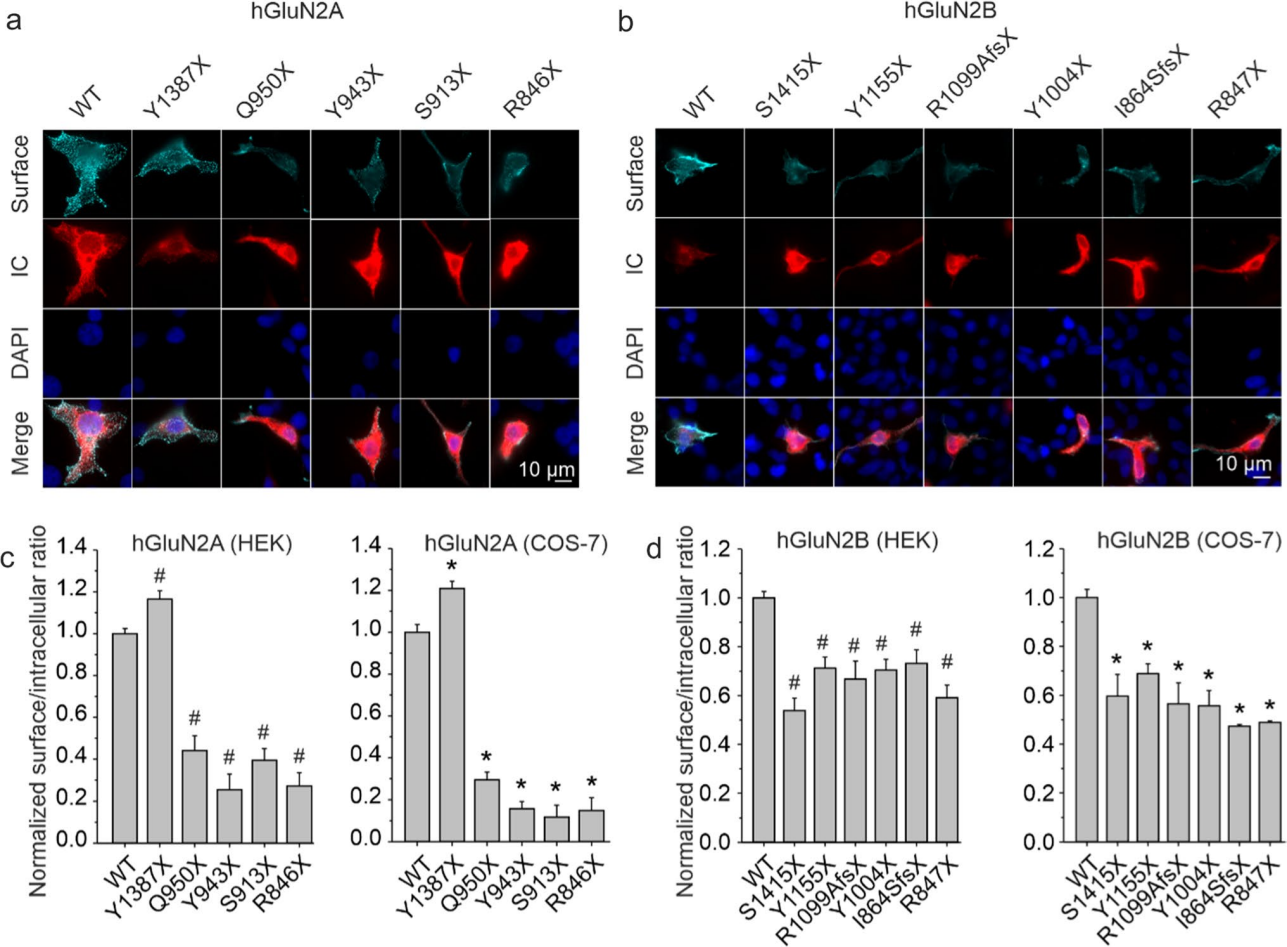
Disease-associated variants of the hGluN2A/B subunits with the truncated CTDs alter the surface expression of NMDARs in HEK and COS-7 cells. HEK and COS˗7 cells were co-transfected with hGluN1 and different WT or variant eGFP-hGluN2A/B subunits. Representative images of surface and intracellular (IC) eGFP-hGluN2A (**a**) and eGFP-hGluN2B (**b**) subunits in HEK cells. DAPI staining was used as an indicator of cell viability. Summary of the relative surface expression of WT or variant eGFP-hGluN2A (*on the left in* **c**) and eGFP-hGluN2B (*on the left in* **d**) subunits measured in HEK cells using fluorescence microscopy. Summary of the relative surface expression of WT or variant eGFP-hGluN2A (*on the right in* **c**) and eGFP-hGluN2B (*on the right in* **d**) subunits measured in COS-7 cells using a quantitative colorimetric assay. All summary data are presented as the mean ± SEM. # marks a significant difference in experiments on HEK cells (data were power transformed and tested by the ANOVA followed by multiple comparisons versus hGluN1/eGFP-hGluN2A/B-WT receptors (Dunnett’s method); hGluN2A: *number of analyzed cells; n* = 268 (WT), 100 (Y1387X), 208 (Q950X), 100 (Y943X), 233 (S913X), and 100 (R846X); hGluN2B: *n* = 533 (WT), 174 (S1415X), 238 (Y1155X), 137 (R1099AfsX51), 205 (Y1004X), 130 (I864SfsX20), and 232 (R847X) from three to four independent experiments). * marks a significant difference in experiments on COS-7 cells (data were tested by the ANOVA followed by multiple comparisons versus hGluN1/eGFP-hGluN2A/B-WT receptors (Dunnett’s method); hGluN2A: *number of independent experiments; n* = 4 (WT), 3 (Y1387X), 4 (Q950X), 3 (Y943X), 3 (S913X), and 3 (R846X); hGluN2B: *n* = 4 (WT), 4 (S1415X), 3 (Y1155X), 4 (R1099AfsX51), 3 (Y1004X), 3 (I864SfsX20), and 3 (R847X)). Relative surface expression was measured in quadruplicate in each experiment

Quantitative analysis of images indicated that the ratio of surface-to-intracellular NMDAR expression was significantly increased for hGluN1/eGFP-hGluN2A(Y1387X) receptors and significantly reduced for hGluN1/eGFP-hGluN2A(R846X), (S913X), (Y943X), and (Q950X) receptors compared to hGluN1/eGFP-hGluN2A-WT receptors when expressed in HEK cells (Fig. 1a, c). The obtained results were confirmed by a colorimetric assay in COS-7 cells, with surface-to-intracellular NMDAR expression significantly increased for hGluN1/eGFP-hGluN2A(Y1387X) receptors and significantly decreased for the rest of the NMDARs containing truncated eGFP-hGluN2A subunits (Fig. 1c). The surface expression of hGluN1/eGFP-hGluN2B(R847X), (I864SfsX20), (Y1004X), (R1099AfsX51), (Y1155X), and (S1415X) receptors was significantly reduced compared to hGluN1/eGFP-hGluN2B-WT receptors as assessed by both approaches, immunofluorescence microscopy on HEK cells, and the colorimetric assay on COS-7 cells (Fig. 1b, d).

To complement the results on NMDAR surface expression, we used patch-clamp electrophysiology to analyze the current density of responses induced by glutamate (1 mM; applied in the continuous presence of 30 µM glycine) in HEK cells transfected with WT and variant subunits. The hGluN1/hGluN2A and hGluN1/hGluN2B receptors with truncated CTD tended to have a reduced current density compared to the corresponding WT receptors. Current densities are influenced by transfection efficiency that can be highly variable between individual cells, and the reduction of current density reached statistical significance only in the case of hGluN1/hGluN2B(I864SfsX20) and (S1415X) receptors (Supplementary Fig. S1).

### Synaptic localization of human NMDARs with truncated CTDs

**Fig. 2:**
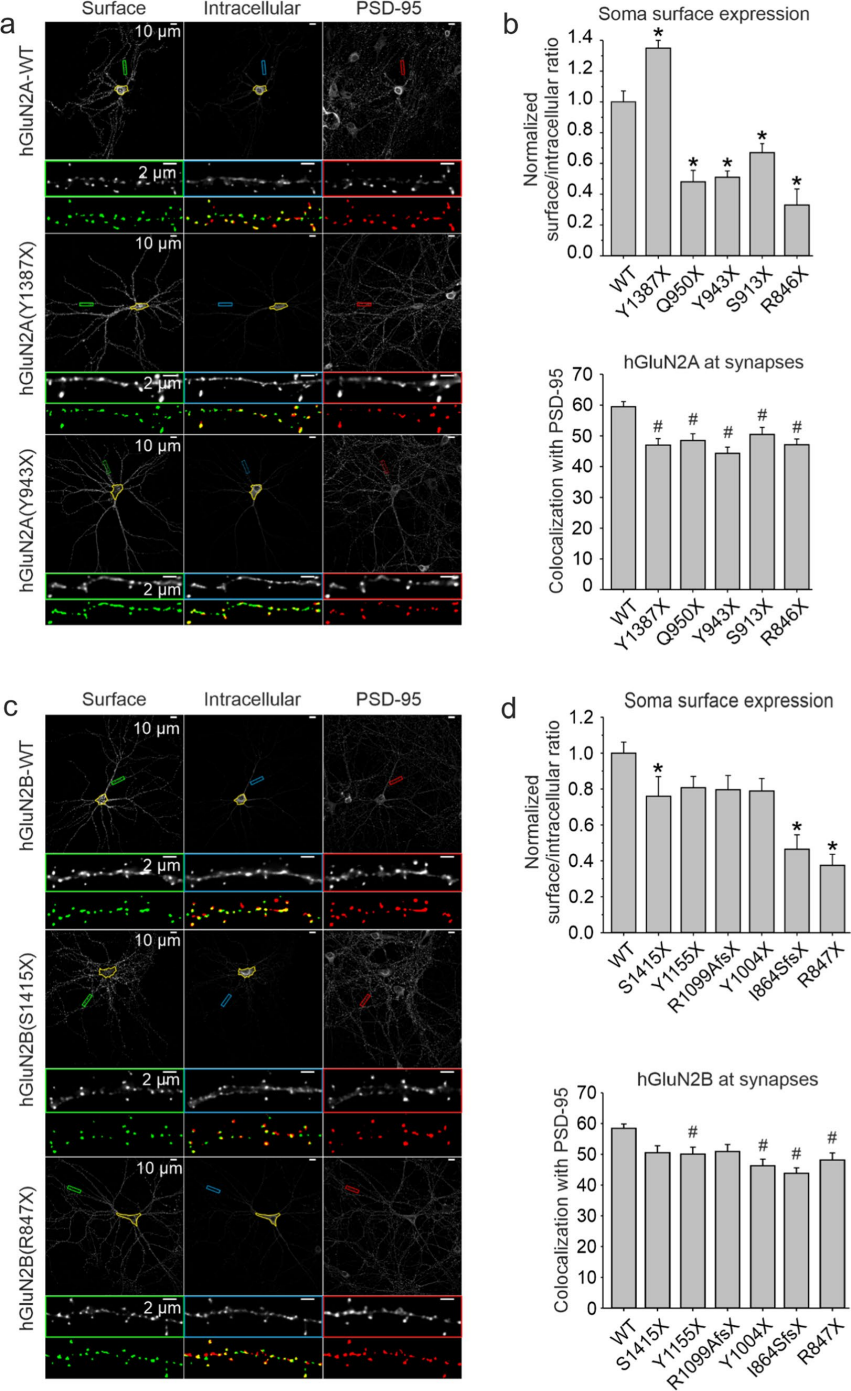
Disease-associated variants of the hGluN2A/B subunits with truncated CTDs alter the surface expression and decrease the synaptic localization of NMDARs in hippocampal neurons. Representative images show surface and intracellular immunofluorescence staining for eGFP-hGluN2A (**a**), eGFP-hGluN2B (**c**), and a postsynaptic marker PSD-95 (**a**, **c**). Areas marked in yellow indicate the somata of neurons and green, blue, and red rectangles indicate secondary dendrites shown below at a higher magnification. The bottom row shows thresholded surface receptors (green) and PSD-95 (red) with their composite image used to calculate the percentage of receptors at synapses. Summary bar graphs show the relative surface expression of eGFP-hGluN2 subunits measured in the somata of hippocampal neurons normalized to WT and the colocalization analysis of the percentage of pixels of the surface eGFP-hGluN2 signal overlapping with the PSD-95 signal for NMDARs containing the eGFP-hGluN2A (**b**) and eGFP-hGluN2B (**d**) subunits. All summary data are presented as the mean ± SEM. * marks a significant difference in receptor surface expression at the soma (data were power transformed and tested by the ANOVA followed by multiple comparisons versus GluN1/eGFP-hGluN2A/B-WT receptors (Dunnett's method); hGluN2A:* n* = 46 (WT), 24 (Y1387X), 24 (Q950X), 24 (Y943X), 24 (S913X), and 24 (R846X); hGluN2B: *n* = 40 (WT), 24 (S1415X), 24 (Y1155X), 24 (R1099AfsX51), 24 (Y1004X), 24 (I864SfsX20), and 24 (R847X)). # marks a significant difference in colocalization of the eGFP-hGluN2 signal with the PSD-95 signal (data were tested by the ANOVA followed by multiple comparisons versus GluN1/eGFP˗hGluN2A/B-WT receptors (Dunnett's method); hGluN2A:* n* = 45 (WT), 24 (Y1387X), 24 (Q950X), 23 (Y943X), 24 (S913X), and 24 (R846X); hGluN2B: *n* = 39 (WT), 25 (S1415X), 27 (Y1155X), 25 (R1099AfsX51), 25 (Y1004X), 25 (I864SfsX20), and 25 (R847X)). Data in b and d are from three independent experiments

Next, we used an immunofluorescence approach to examine the synaptic localization of NMDARs with truncated CTDs of GluN2A/B subunits in primary hippocampal neurons. We introduced the WT or variant eGFP-tagged hGluN2A/B subunits into primary mouse hippocampal neurons using lentiviral particles (see Methods). In this model, the surface expression of the introduced eGFP-hGluN2A/B subunits was therefore fully dependent on the expression of native GluN1 subunits. First, we used immunofluorescence microscopy to detect the surface and intracellular protein levels of eGFP-tagged NMDARs in the soma of neurons. Quantitative analysis of images of cultured hippocampal neurons confirmed our results obtained from HEK and COS-7 cells. The results suggested reduced surface expression for all the studied NMDARs containing truncated eGFP-hGluN2A/B subunits, except for GluN1/eGFP-hGluN2A(Y1387X) receptors, whose surface expression was significantly increased. For eGFP-hGluN2A(Q950X), (Y943X), (S913X), (R846X) and eGFP-hGluN2B (S1415X), (I864SfsX20), (R847X) we observed significantly reduced levels of surface expression (Fig. 2a–d).

To characterize the synaptic localization of NMDARs containing WT or truncated GluN2A and GluN2B subunits, we used immunofluorescence to stain postsynaptic scaffold protein PSD-95, and determined the degree of colocalization of the surface eGFP-hGluN2A/B and the PSD-95 signals (see Methods). The percentage of the surface eGFP-hGluN2A/B pixels overlapping with PSD-95 indicated a reduced level of synaptic localization for all NMDARs containing truncated eGFP-hGluN2A/B subunits, possibly due to the loss of the ESDV PDZ domain-binding motif located at the very end of the GluN2A/B CTDs. For GluN1/eGFP-hGluN2B(S1415X) and (R1099AfsX51) receptors, the reduction in synaptic localization was not significant (Fig. 2b, d). In addition, to assess the possible effect of variant subunits on the number of dendritic spines, we counted the total number of puncta of surface receptors and PSD-95 per 20 µm of secondary dendrites, with no significant differences between neurons expressing the WT and the truncated eGFP-hGluN2 subunits (data not shown).

### Consequences of disease-associated variants in the CTD for NMDAR function

**Fig. 3:**
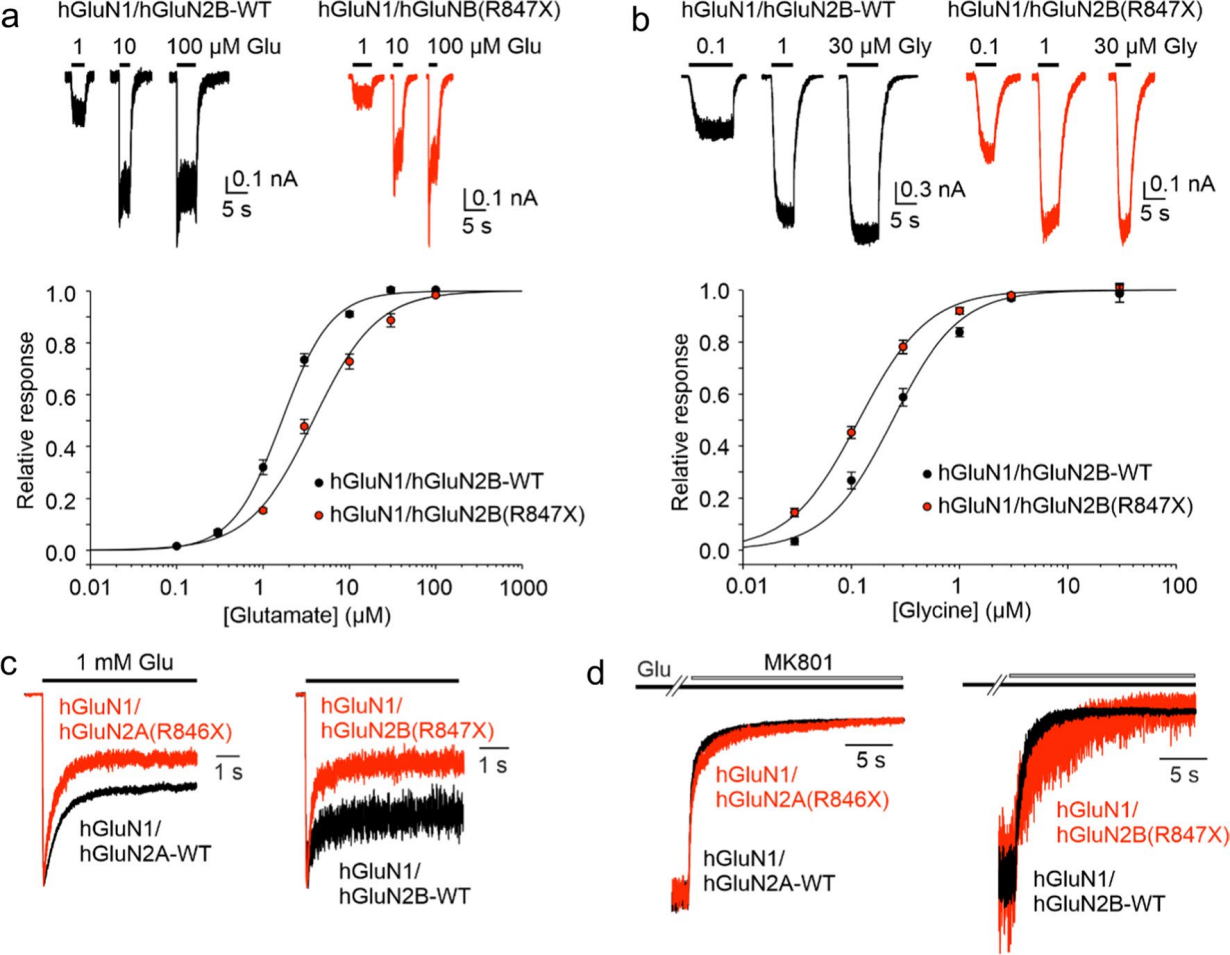
Disease-associated variants of the hGluN2A/B subunits with truncated CTDs alter NMDAR functional properties. **a** Representative recordings of responses to 1, 10, and 100 µM glutamate applications made in the presence of 30 µM glycine are shown for HEK cells expressing hGluN1/hGluN2B-WT or hGluN1/hGluN2B(R847X) receptors. The plot shows peak dose–response relationship for glutamate activation of hGluN1/hGluN2B-WT (black circles) and hGluN1/hGluN2B(R847X) receptors (red circles). Data points for the glutamate responses from each cell were fitted by *Eq. *2. The smooth curve represents the mean of data from *n* = 9 cells for hGluN1/hGluN2B-WT and *n* = 5 cells for hGluN1/hGluN2B(R847X) receptors. **b** Representative recordings of responses to 0.1, 1, and 30 µM glycine applications made in the presence of 1 mM glutamate are shown for HEK cells expressing hGluN1/hGluN2B-WT or hGluN1/hGluN2B(R847X) receptors. The plot shows peak dose–response relationship for glycine activation of hGluN1/hGluN2B-WT (black circles) and hGluN1/hGluN2B(R847X) receptors (red circles). Data points for the glycine responses from each cell were fitted by *Eq. *2. The smooth curve represents the mean of data from *n* = 13 cells for hGluN1/hGluN2B-WT and *n* = 7 cells for hGluN1/hGluN2B(R847X) receptors. Data points in (**a**) and (**b**) represent mean agonist-induced effect ± SEM. **c** Representative currents induced in hGluN1/hGluN2A-WT or hGluN1/hGluN2A(R846X) receptors and hGluN1/hGluN2B-WT or hGluN1/hGluN2B(R847X) receptors by fast application of 1 mM glutamate in the continuous presence of 30 µM glycine are shown normalized with respect to the maximal response. **d** Representative responses of hGluN1/hGluN2A-WT or hGluN1/hGluN2A(R846X) receptors and hGluN1/hGluN2B-WT or hGluN1/hGluN2B(R847X) receptors showing the onset of MK-801 (1 µM) inhibition of normalized currents induced by application of 1 mM glutamate in the continuous presence of 30 µM glycine

We next used patch-clamp electrophysiology to characterize the functional effects of the selected disease-associated hGluN2A/B subunit variants with CTD truncation (Table 1) co-expressed with the hGluN1 subunit in HEK cells. The analysis of glutamate potency for truncated human NMDARs indicated that the EC_50_ was significantly increased for hGluN1/hGluN2B(R847X) receptors. In contrast, for NMDARs containing the other hGluN2B CTD truncations and for all of the NMDARs containing hGluN2A CTD truncations, glutamate potency was not significantly different from receptors containing hGluN2A/B-WT subunits (Fig. 3a, Table 2). A similar analysis of glycine potency indicated that the EC_50_ was significantly diminished for hGluN1/hGluN2B(I864SfsX20) and hGluN1/hGluN2B(R847X) receptors while for the remaining truncated hGluN2A and hGluN2B variants glycine potency was not significantly different from the receptors containing hGluN2A/B-WT subunits (Fig. 3b, Table 2).

**Table 2:** Effects of hGluN2A and hGluN2B CTD truncations on NMDAR agonist potency

Subunit	Variant	Glutamate			Glycine		
		EC_50_ (μM)	*h*	*n*	EC_50_ (μM)	*h*	*n*
hGluN2A	WT	5.0 ± 0.3	1.09 ± 0.02	6	1.50 ± 0.17	1.20 ± 0.07	6
hGluN2A	Y1387X	4.3 ± 0.4	1.24 ± 0.07	5	1.50 ± 0.21	1.29 ± 0.29	5
hGluN2A	Q950X	7.0 ± 0.7	1.29 ± 0.06	7	1.29 ± 0.16	1.30 ± 0.10	5
hGluN2A	Y943X	7.3 ± 0.7	1.27 ± 0.07	11	1.85 ± 0.16	1.43 ± 0.08	5
hGluN2A	S913X	6.0 ± 0.9	1.13 ± 0.08	5	1.85 ± 0.05	1.61 ± 0.07	5
hGluN2A	R846X	5.7 ± 0.5	1.04 ± 0.08	5	0.72 ± 0.05	1.30 ± 0.14	6
hGluN2B	WT	1.7 ± 0.1	1.33 ± 0.03	9	0.24 ± 0.02	1.41 ± 0.08	13
hGluN2B	S1415X	1.9 ± 0.3	1.27 ± 0.08	4	0.26 ± 0.02	1.46 ± 0.06	5
hGluN2B	Y1155X	1.7 ± 0.2	1.32 ± 0.03	7	0.26 ± 0.02	1.29 ± 0.17	5
hGluN2B	R1099AfsX51	1.6 ± 0.1	1.39 ± 0.05	7	0.28 ± 0.01	1.39 ± 0.07	5
hGluN2B	Y1004X	1.5 ± 0.1	1.30 ± 0.05	5	0.19 ± 0.04	1.51 ± 0.19	4
hGluN2B	I864SfsX20	1.5 ± 0.3	1.33 ± 0.04	5	0.11 ± 0.01*	1.40 ± 0.10	6
hGluN2B	R847X	3.9 ± 0.3*	1.37 ± 0.05	5	0.12 ± 0.01*	1.36 ± 0.09	7

Values are the mean ± SEM

Values that showed a significant difference from wild type are in bold

*Significant difference (ANOVA followed by multiple comparisons versus WT hGluN1/hGluN2A/B receptors (Dunnett’s method); statistical analysis performed for logEC_50_ values)

Next, we evaluated the effects of hGluN2A/B CTD truncations on NMDAR desensitization, a time-dependent decline of responses (see Eq. 3 for definition). Receptors were activated by a saturating concentration of glutamate (1 mM) in the continuous presence of glycine (30 µM). The analysis of the responses indicated that hGluN1/hGluN2A-WT and hGluN1/hGluN2B-WT receptors desensitized on average by 42% and 19%, respectively (Fig. 3c). Desensitization was significantly increased in receptors with the most extensive CTD truncation (hGluN1/hGluN2A(R846X), hGluN1/hGluN2B(I864SfsX20), and hGluN1/hGluN2B(R847X) receptors) (Fig. 3c, Table 3). Receptors with shorter hGluN2A or hGluN2B CTD truncations exhibited the degree of desensitization similar to WT receptors. Increased desensitization of receptors containing hGluN2A/B subunits with CTD truncations was partly due to the increased value of the desensitization rate constant (*k*_d_) that was observed for hGluN1/hGluN2A(R846X), hGluN1/hGluN2B(I864SfsX20), and hGluN1/hGluN2B(R847X) receptors when compared to the value of *k*_d_ determined for the corresponding WT receptors. The value of the resensitization rate constant (*k*_r_) was increased in hGluN1/hGluN2A(Q950X), hGluN1/hGluN2A(R846X), and hGluN1/hGluN2B(I864SfsX20) receptors when compared to the value of *k*_r_ determined for the corresponding WT receptors (Table 3).

**Table 3:** Effects of hGluN2A and hGluN2B CTD truncations on NMDAR desensitization and *P*_o_

Subunit	Variant	Desensitization				*P*_o_	
		%	*k*_d_	*k*_r_	*n*	%	*n*
hGluN2A	WT	42 ± 4	0.47 ± 0.09	0.59 ± 0.04	7	19.1 ± 1.0	7
hGluN2A	Y1387X	33 ± 3	0.28 ± 0.05	0.54 ± 0.04	5	20.9 ± 1.4	5
hGluN2A	Q950X	46 ± 6	0.67 ± 0.20	**0.66 ± 0.06**^**#**^	5	23.3 ± 3.8	5
hGluN2A	Y943X	30 ± 3	0.29 ± 0.05	0.65 ± 0.05	7	21.7 ± 2.7	6
hGluN2A	S913X	30 ± 3	0.25 ± 0.06	0.54 ± 0.09	6	17.9 ± 2.3	6
hGluN2A	R846X	62 ± 5*	**2.50 ± 0.52**^**#**^	**1.37 ± 0.21**^**#**^	5	5.8 ± 1.1*	5
hGluN2B	WT	19 ± 3	0.52 ± 0.19	1.75 ± 0.43	9	9.2 ± 0.8	9
hGluN2B	S1415X	21 ± 3	0.49 ± 0.14	1.84 ± 0.50	5	8.0 ± 1.1	5
hGluN2B	Y1155X	14 ± 2	0.30 ± 0.06	2.40 ± 0.71	6	7.4 ± 1.2	6
hGluN2B	R1099AfsX51	22 ± 2	0.55 ± 0.16	1.86 ± 0.42	5	8.8 ± 0.6	5
hGluN2B	Y1004X	18 ± 3	0.77 ± 0.58	2.36 ± 1.31	5	7.3 ± 0.8	5
hGluN2B	I864SfsX20	**48 ± 5**^**#**^	**4.23 ± 0.91**^**#**^	**4.57 ± 0.89**^**#**^	5	2.5 ± 0.6*	5
hGluN2B	R847X	**54 ± 3**^**#**^	**2.32 ± 0.37**^**#**^	2.32 ± 0.37	6	3.2 ± 0.4*	6

Values are the mean ± SEM

Values that showed a significant difference from wild type are in bold

*Significant difference (ANOVA followed by multiple comparisons versus WT hGluN1/hGluN2A/B receptors (Dunnett’s method))

^#^Significant difference (data were power transformed and tested using the ANOVA followed by multiple comparisons versus WT hGluN1/hGluN2A/B receptors (Dunnett's method))

We also characterized the effect of disease-associated hGluN2A/B CTD truncations on the channel open probability (*P*_o_) of variant NMDARs. NMDARs were activated by a saturating concentration of glutamate (1 mM) and the time course of channel inhibition by 1 µM MK-801 was fitted by a kinetic model that allowed the determination of the *P*_o_ (see Methods). Superimposed traces (Fig. 3d) show examples of the onset of MK-801 inhibition, which is decelerated in hGluN1/hGluN2A(R846X) and hGluN1/hGluN2B(R847X) receptors compared to the corresponding WT receptors. The analysis indicated that the *P*_o_ decreased significantly from 19.1% in hGluN1/hGluN2A-WT receptors to 5.8% in hGluN1/hGluN2A(R846X) receptors. Similarly, the *P*_o_ decreased from 9.2% determined in hGluN1/hGluN2B-WT receptors to 2.5% in hGluN1/hGluN2B(I864SfsX20), and 3.2% in hGluN1/hGluN2B(R847X) receptors (Table 3). Together, these data indicate that disease-associated de-novo nonsense and frame-shift variants in the hGluN2A/B CTD can alter even NMDAR properties primarily associated with other receptor domains, such as desensitization and *P*_o_, properties associated with the TMD, or agonist affinity, a property primarily associated with the ABD.

### Effects of disease-associated hGluN2A/B variants with CTD truncations on NMDAR steroid sensitivity

**Fig. 4:**
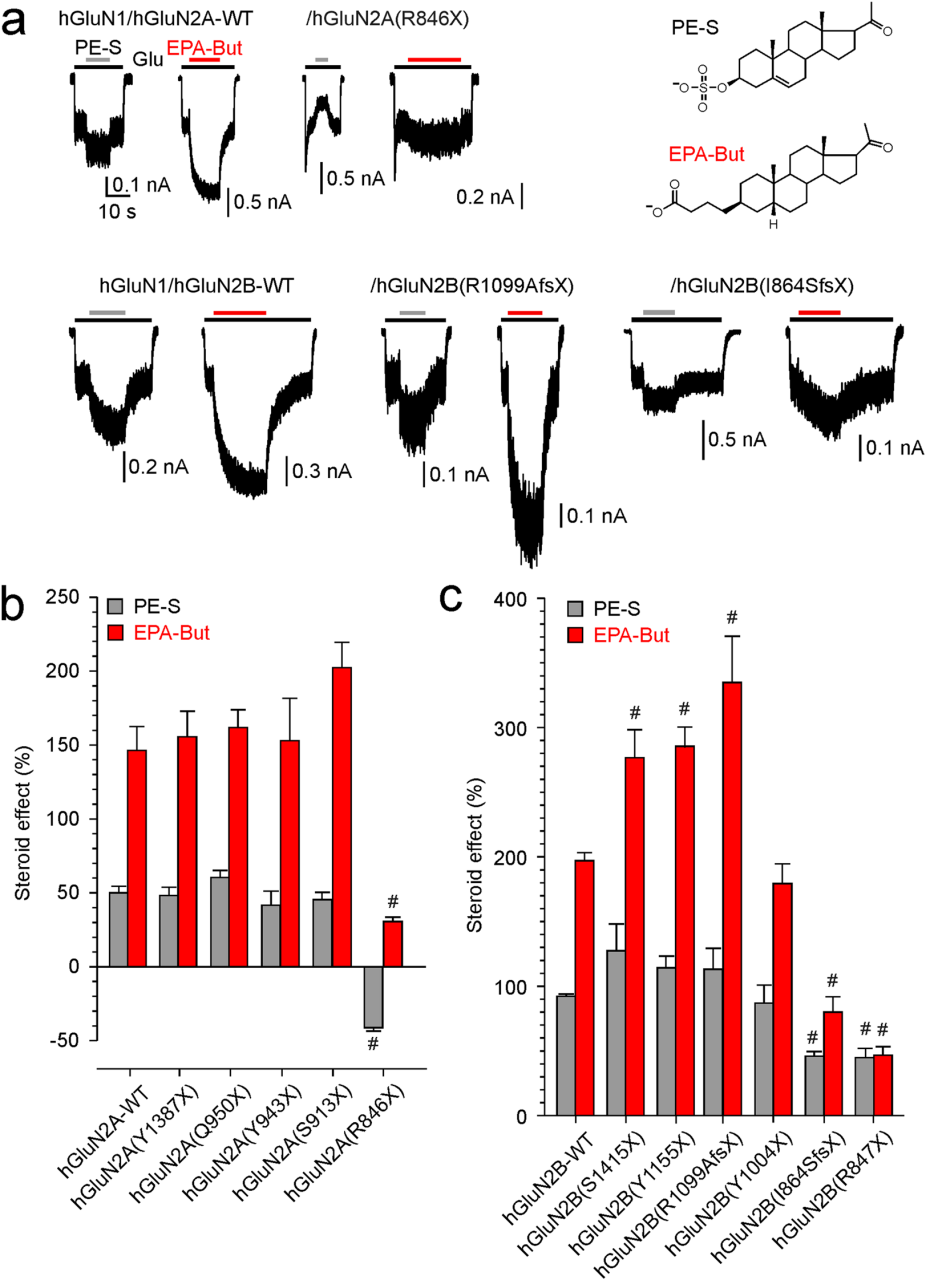
Effects of PE-S and EPA-But on NMDARs with disease-associated variants of hGluN2A or hGluN2B subunits with truncated CTDs. **a** Representative recordings of responses to glutamate (3 µM for hGluN1/hGluN2A and 1 µM for hGluN1/hGluN2B receptors) made before and in the presence of PE-S (100 µM) or EPA-But (15 µM) are shown for hGluN1/hGluN2A-WT, hGluN1/hGluN2A(R846X), and hGluN1/hGluN2B-WT, hGluN1/hGluN2B(R1099AfsX51), and hGluN1/hGluN2B(I864SfsX20) receptors. Inset shows the structures of neurosteroids used. **b** Steroid effects on glutamate responses induced in receptors with CTD truncation in hGluN2A. **c** Steroid effects on glutamate responses induced in receptors with CTD truncation in hGluN2B. Data represent mean steroid potentiation ( +) or inhibition (−) in % ± SEM (*n* = 5–66). # marks receptors with significantly altered steroid (PE-S/EPA-But) sensitivity (data were power transformed and tested using the ANOVA followed by multiple comparisons versus hGluN1/hGluN2A/B-WT receptors (Dunnett’s method))

Endogenous steroids are potent allosteric modulators of NMDAR activity [49, 50]. Therefore, we next investigated the sensitivity of truncated receptors to PE-S. This naturally occurring steroid has a subunit-dependent positive allosteric effect at the NMDARs (with a preference for receptors containing GluN2A/B subunits over GluN2C/D subunits) [51, 52]. We also tested EPA-But, a synthetic steroid with a positive allosteric effect whose binding sites at the NMDAR are partially non-overlapping with those for PE-S [27]. Figure 4a, b shows that PE-S (100 µM) and EPA-But (15 µM) potentiate hGluN1/hGluN2A/B-WT receptors and, to different extents, also modulate the activity of receptors with truncated hGluN2A or hGluN2B CTDs. Specifically, receptors with a fully truncated hGluN2A subunit (hGluN1/hGluN2A(R846X)) were less potentiated by EPA-But and the potentiating effect of PE-S was inverted to inhibition (Fig. 4a, b). Receptors containing the hGluN2B subunit truncated at the more distal sites (hGluN2B(S1415X), hGluN2B(Y1155X), and hGluN2B(R1099AfsX51)) were more potentiated by EPA-But while the effect of PE-S was similar as in hGluN1/hGluN2B-WT receptors. Receptors with truncations at sites proximal to the M4 transmembrane helix (hGluN1/hGluN2B(I864SfsX20) and hGluN2B(R847X) were less sensitive to the potentiating effect of both PE-S and EPA-But (Fig. 4a, c). These results suggest that hGluN2A/B CTD truncation affects the degree of the modulatory effect of PE-S and EPA-But at NMDARs and that the relative modulatory effects of these two steroids differ depending on the site of the CTD truncation.

### Palmitoylation at the GluN2B CTD controls NMDAR steroid sensitivity

**Fig. 5:**
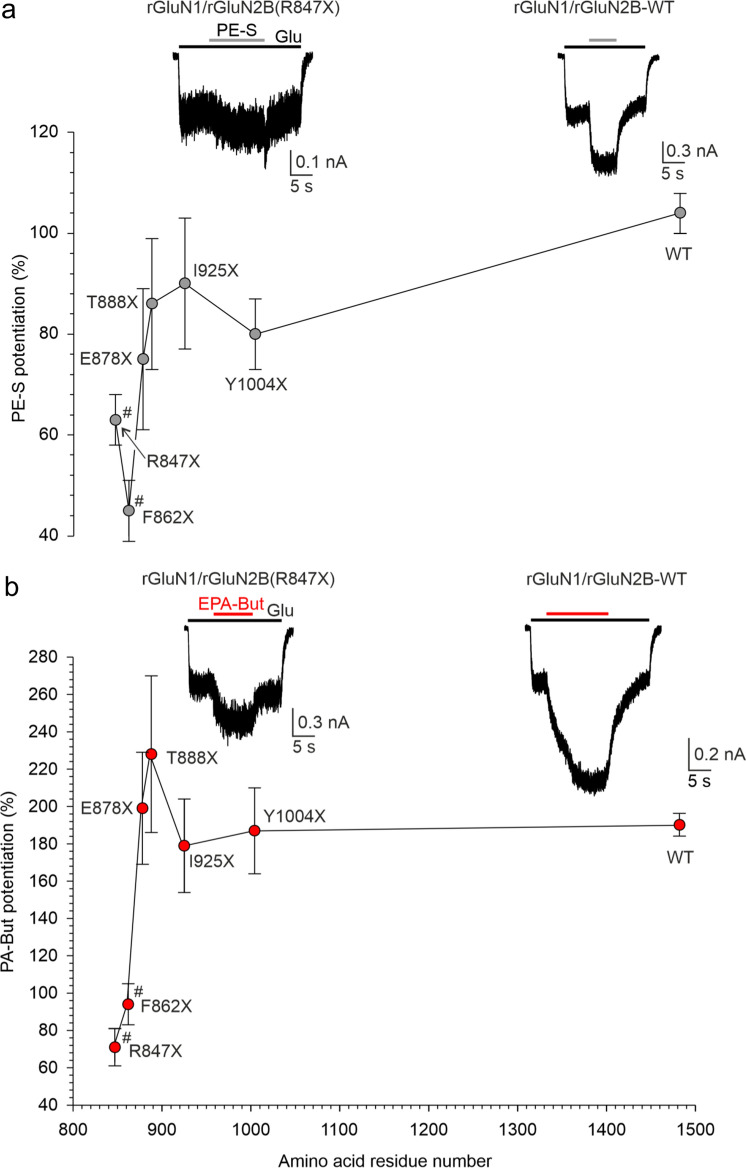
Juxtamembrane region of the rGluN2B CTD is critical for controlling NMDAR steroid sensitivity. Examples of traces obtained from HEK cells expressing rGluN1/rGluN2B-WT and rGluN1/rGluN2B(R847X) receptors. PE-S (100 µM) (**a**) and EPA-But (15 µM) (**b**) were applied in the presence of glutamate (1 µM). The graphs show the relative degree of potentiation induced by the steroid (PE-S, gray symbols; EPA-But, red symbols) in receptors with the progressively truncated rGluN2B CTD. Data represent mean steroid potentiation in % ± SEM (*n* = 5–161). # marks receptors with significantly altered steroid (PE-S/EPA-But) sensitivity (data were power transformed and tested by the ANOVA followed by multiple comparisons versus rGluN1/rGluN2B-WT receptors (Dunnett's method))

We next set out to investigate the mechanisms by which CTD regions regulate NMDAR sensitivity to potentiating steroids. For better comparison with our previous work where we had described the role of the GluN2B CTD in regulating NMDAR sensitivity to inhibitory steroids [10], we used recombinant rat NMDARs expressed in HEK cells. To determine the location within the GluN2B CTD that controls receptor steroid sensitivity, we analyzed the effect of PE-S and EPA-But on receptors containing the rGluN2B subunit with a progressively shortened CTD, co-expressed together with rGluN1-1a (rGluN1) subunit. Responses evoked in rGluN1/rGluN2B-WT receptors by 1 µM glutamate were potentiated by 100 μM PE-S (104 ± 3%; *n* = 161) and 15 µM EPA-But (190 ± 6%; *n* = 126) to the extent that was not significantly different from their human isoforms. Figure 5a, b shows that smaller CTD truncations (rGluN1/rGluN2B(Y1004X) to rGluN1/rGluN2B(E878X) receptors) had only a small effect on the potentiation of responses to 1 µM glutamate by PE-S (100 µM) or EPA-But (15 µM). However, receptors with more extensive CTD truncations (rGluN1/rGluN2B(F862X) and rGluN1/rGluN2B(R847X)) were less potentiated by either steroid compared to the rGluN1/rGluN2B-WT receptors. These results indicate that a stretch of amino acid residues located in the juxtamembrane region of the GluN2B CTD is critical for controlling NMDAR sensitivity to both PE-S and EPA-But, and that the activity of both steroids is affected to a similar extent in receptors with the truncated CTD of rGluN2B subunit.

**Fig. 6:**
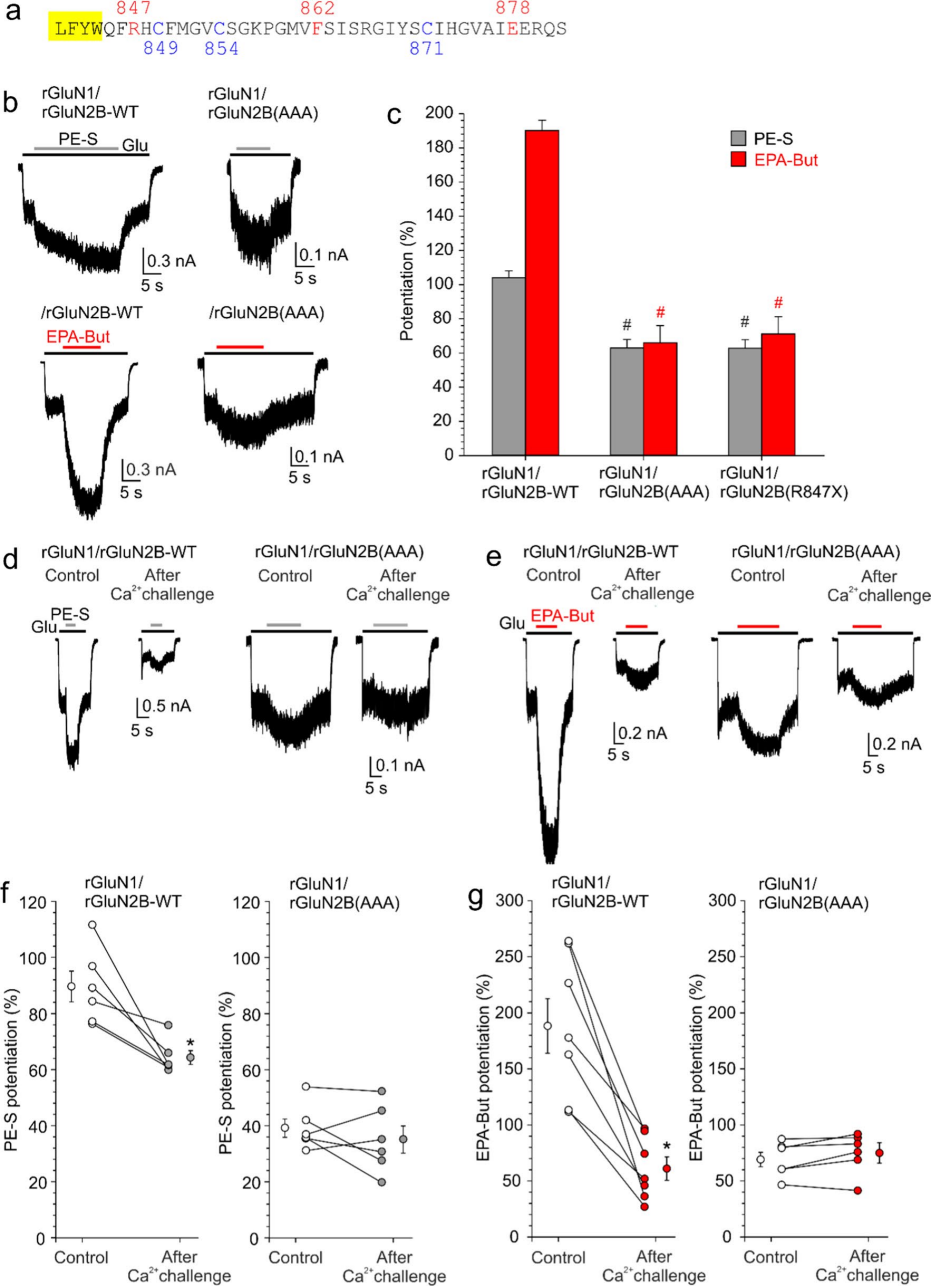
Palmitoylation of the rGluN2B CTD controls NMDAR steroid sensitivity. **a** Amino acid sequence of a portion of the M4 helix and the juxtamembrane region of the rGluN2B CTD. The membrane region is highlighted in yellow. The sites at which the CTD was truncated in Fig. 5 are shown in red (labeled above). Cysteines that can be palmitoylated are shown in blue (labeled below). **b** Examples of responses induced in rGluN1/rGluN2B-WT and rGluN1/rGluN2B(AAA) receptors with the cysteine palmitoylation sites in the rGluN2B subunit changed to alanine (C849A, C854A, C871A). **c** The bar graph shows the relative degree of PE-S (100 µM) and EPA-But (15 µM) potentiation of responses to 1 µM glutamate. Data represent mean steroid-induced potentiation in % ± SEM. # (black/red) marks significantly altered steroid (PE-S/EPA-But) sensitivity determined in rGluN1/rGluN2B(AAA) and rGluN1/rGluN2B(R847X) receptors when compared to rGluN1/rGluN2B-WT receptors (data were power transformed and tested by the ANOVA followed by multiple comparisons versus rGluN1/rGluN2B-WT receptors (Dunnett's method)); PE-S: *n* = 8–20; EPA-But: *n* = 7–19). **d**, **e** Examples of responses obtained from HEK cells expressing rGluN1/rGluN2B-WT or rGluN1/rGluN2B(AAA) receptors. PE-S (100 μM) (**d**) and EPA-But (15 µM) (**e**) were applied in the presence of 1 μM glutamate. Gray/red and black bars represent the duration of the steroid and the agonist application, respectively, in the continuous presence of 0.2 mM [Ca^2+^]_*o*_ before (Control) and after the application of 1 mM glutamate in the presence of 2 mM [Ca^2+^]_*o*_ for 50 s (After Ca^2+^ challenge). **f**, **g** Graphs represent the potentiating effect of PE-S (100 μM) (**f**) and EPA-But (15 μM) (**g**) on control responses (Control, open symbols) and on responses recorded following the Ca^2+^ challenge (After Ca^2+^ challenge; gray/red symbols). Data points with error bars represent mean steroid-induced potentiation in % ± SEM. * indicates significantly altered steroid sensitivity. Data were statistically analyzed using paired t tests (rGluN1/rGluN2B-WT PE-S: *p* = 0.011; rGluN1/rGluN2B(AAA) PE-S: *p* = 0.355 (**f**); rGluN1/rGluN2B-WT EPA-But: *p* = 0.001; rGluN1/rGluN2B(AAA) EPA-But: *p* = 0.122 (**g**))

The critical region of the rGluN2B CTD (R847–E878) contains three cysteine residues, C849, C854, and C871, that can be palmitoylated [53] (Fig. 6a). To test whether the NMDAR sensitivity to potentiating steroids is affected by palmitoylation, we generated rGluN2B subunit where all three cysteines (C849, C854, and C871) were changed to non-palmitoylable alanine (rGluN2B(AAA) subunit). Compared to rGluN1/rGluN2B-WT receptors, rGluN1/rGluN2B(AAA) receptors were less potentiated by PE-S (100 µM) and EPA-But (15 µM) (Fig. 6b, c). The steroid-induced potentiation of responses to 1 µM glutamate in rGluN1/rGluN2B(AAA) receptors was not significantly different from the potentiation of receptors with the full CTD truncation (rGluN1/rGluN2B(R847X) receptors) (Fig. 6c).

Recently, we have shown that a transient rise in the intracellular Ca^2+^ concentration ([Ca^2+^]_*i*_) can induce NMDAR depalmitoylation with long-lasting consequences for the receptor sensitivity to inhibitory steroids [10]. Therefore, we next examined the effect of increased [Ca^2+^]_*i*_ on the degree of PE-S and EPA-But potentiation of NMDARs. The experimental protocol consisted of an assessment of the steroid effect in the presence of low extracellular Ca^2+^ concentration ([Ca^2+^]_*o*_ = 0.2 mM) before (control) and after (test) the application of 1 mM glutamate in the presence of 2 mM [Ca^2+^]_*o*_ for 50 s (Ca^2+^ challenge). We have shown earlier, using quantitative ratiometric Ca^2+^ imaging, that the Ca^2+^ challenge induces a robust increase in the [Ca^2+^]_*i*_ [10]. This is expected since NMDAR ion channels are highly permeable to Ca^2+^ [54] and the buffering capacity of 5 mMEGTA in the intracellular solution is insufficient to fully buffer the Ca^2+^ influx, since EGTA is a relatively slow Ca^2+^ chelator [55]. As shown in Fig. 6d–g, responses of rGluN1/rGluN2B-WT receptors to 1 µM glutamate were robustly potentiated by PE-S (100 µM) and EPA-But (15 µM). This potentiation was significantly reduced in test responses recorded after the Ca^2+^ challenge compared to control responses prior to the Ca^2+^ challenge. The potentiation did not substantially recover during the next 5 min of recording. Consistent with Ca^2+^-induced NMDAR inactivation [56, 57], the maximal amplitude of the test responses was, on average, 30.6 ± 1.7% (*n* = 60) of the amplitude of the control responses. In contrast, experiments performed with rGluN1/rGluN2B(AAA) receptors showed that the small steroid potentiating effect in these receptors was not further altered by the Ca^2+^ challenge. These results suggest that palmitoylation is a key mechanism controlling the rGluN1/rGluN2B receptor sensitivity to steroids. The Ca^2+^ challenge likely induces depalmitoylation of cysteines at the juxtamembrane region of the GluN2B CTD with consequences for the receptor function and pharmacology.

### Mechanisms controlling steroid sensitivity of NMDARs containing the GluN2A subunit

**Fig. 7:**
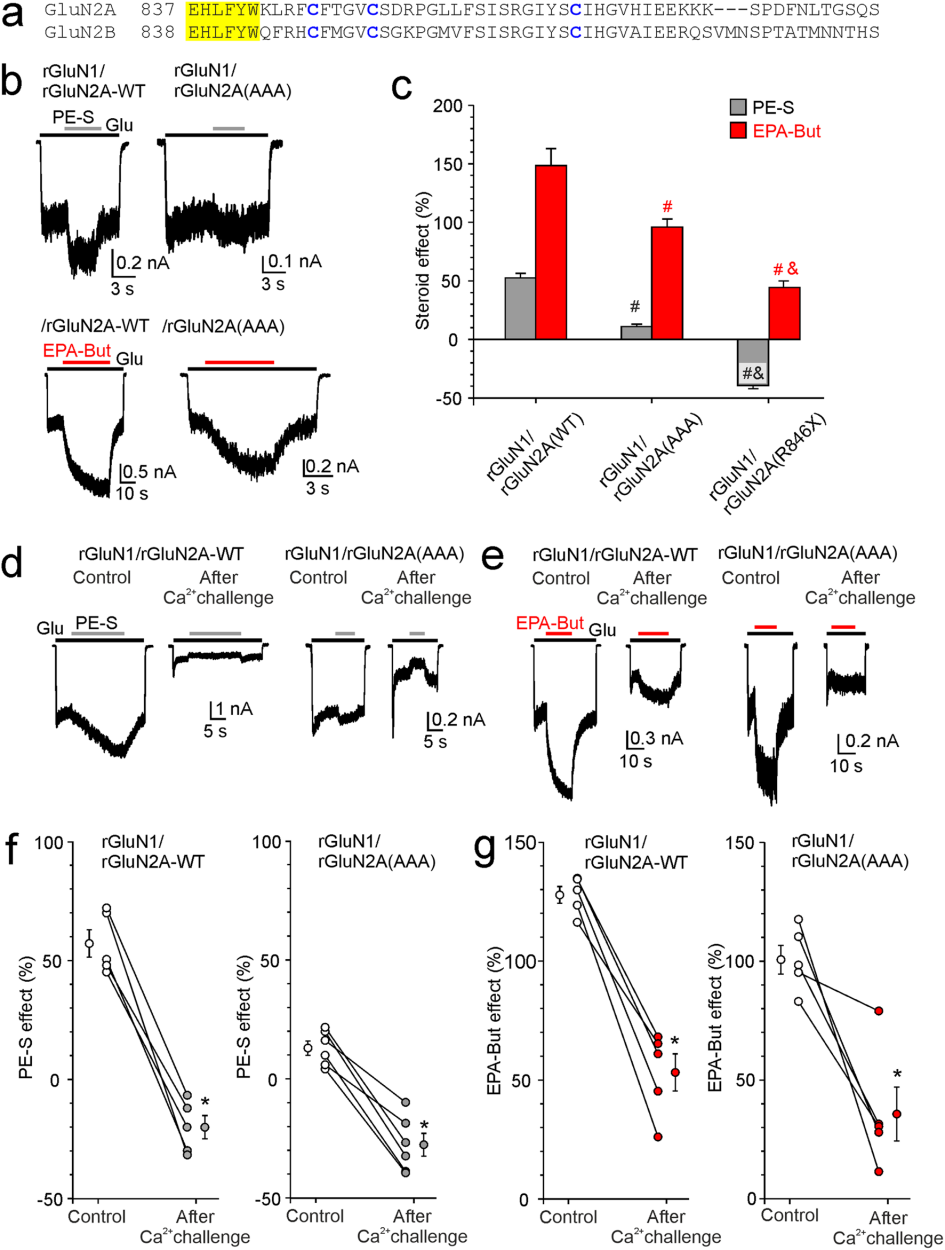
Multiple mechanisms control NMDAR steroid sensitivity via the rGluN2A CTD. **a** Amino acid sequence alignment of the portion of the M4 domain and the proximal region of the CTD across rat GluN2A and GluN2B subunits. The numbering is for the mature protein. The portion of the M4 helix facing intracellularly (yellow) and the palmitoylable cysteines (blue) are shown. **b** Examples of responses of rGluN1/rGluN2A-WT and rGluN1/rGluN2A(AAA) receptors with the cysteine palmitoylation sites in the CTD of rGluN2A subunit changed to alanines (C848A, C853A, C870A). **c** The bar graph shows the relative degree of PE-S (100 µM) and EPA-But (15 µM) modulation of responses to 3 µM glutamate. Data represent the mean steroid-induced modulation in % ± SEM (*n* = 7–14). # (black/red) symbols indicate significantly altered steroid (PE-S/EPA-But) sensitivity determined in rGluN1/rGluN2A(AAA) and rGluN1/rGluN2A(R846X) receptors when compared to rGluN1/rGluN2A-WT receptors. Data were power transformed and tested using the ANOVA followed by multiple comparisons versus rGluN1/rGluN2A-WT receptors (Dunnett’s method). & (black/red) symbols indicate significantly different steroid (PE-S/EPA-But) sensitivity determined in rGluN1/rGluN2A(AAA) compared to rGluN1/rGluN2A(R846X) receptors. Data were power transformed and tested using the ANOVA followed by pairwise comparisons (Duncan method). **d**, **e** Examples of responses obtained from HEK cells expressing rGluN1/rGluN2A-WT and rGluN1/rGluN2A(AAA) receptors. PE-S (100 μM) (**d**) or EPA-But (15 µM) (**e**) were applied in the presence of 3 μM glutamate. Gray/red and black bars represent the duration of the steroid and the agonist application, respectively, in the continuous presence of 0.2 mM [Ca^2+^]_*o*_ before (Control) and after the application of 1 mM glutamate in the presence of 2 mM [Ca^2+^]_*o*_ for 50 s (After Ca^2+^ challenge). **f**, **g** Graphs represent the modulatory effect of PE-S (100 μM) (**f**) and EPA-But (15 μM) (**g**) on control responses (Control, open symbols) and on responses recorded following the Ca^2+^ challenge (After Ca^2+^ challenge; gray/red symbols). Data points with error bars represent the mean steroid-induced modulation in % ± SEM. * indicates significantly altered steroid sensitivity. Data were analyzed using paired t tests (rGluN1/rGluN2A-WT PE-S: *p* < 0.001; rGluN1/rGluN2A(AAA) PE-S: *p* < 0.001 (**f**); rGluN1/rGluN2A-WT EPA-But: *p* < 0.001; rGluN1/rGluN2A(AAA) EPA-But: *p* = 0.013) (**g**))

The M4 helices and the initial segment of the GluN2A and GluN2B CTD share a high degree of homology, including the position of the three palmitoylable cysteines (Fig. 7a). Next, we analyzed the mechanisms that control the steroid sensitivity of NMDARs containing the rGluN2A subunit. Figure 7b, c shows the results for NMDARs with the cysteines C848, C853, and C870 in the CTD of rGluN2A subunit changed to non-palmitoylable alanines (rGluN2A(AAA) subunit). Similarly to receptors containing the rGluN2B subunit, the potentiation of responses by PE-S (100 µM) and EPA-But (15 µM) was reduced in rGluN1/rGluN2A(AAA) receptors compared to rGluN1/rGluN2A-WT receptors. However, in contrast to receptors containing rGluN2B, the extent of EPA-But potentiation was further lowered in receptors with a completely truncated rGluN2A CTD (rGluN1/rGluN2A(R846X) receptors) and these receptors were inhibited by PE-S (compare Figs. 6c and 7c), consistent with results at hGluN1/hGluN2A(R846X) receptors (Fig. 4b). We subsequently evaluated the effect of the Ca^2+^ challenge on the steroid potentiation of rGluN1/rGluN2A-WT and rGluN1/rGluN2A(AAA) receptors. The Ca^2+^ challenge reduced the steroid potentiation of rGluN1/rGluN2A-WT receptors, similar to receptors containing rGluN2B-WT subunit (compare Figs. 6f, g and 7f, g). In contrast to receptors containing rGluN2B(AAA) subunit, for receptors containing rGluN2A(AAA) subunit, the Ca^2+^ challenge further reduced the EPA-But potentiation of glutamate responses and the effect of PE-S in these receptors was turned to inhibition (compare Figs. 6f, g and 7f, g). These results suggest that apart from palmitoylation, which plays a role in receptors containing rGluN2A and rGluN2B subunits, an additional palmitoylation-independent (but Ca^2+^-dependent) mechanism exists in receptors containing the rGluN2A subunit. We have not investigated this mechanism in any further detail in this study.

### Potentiating effect of PE-S and EPA-But is differentially affected by the CTDs from GluN1 and GluN2B subunits

**Fig. 8:**
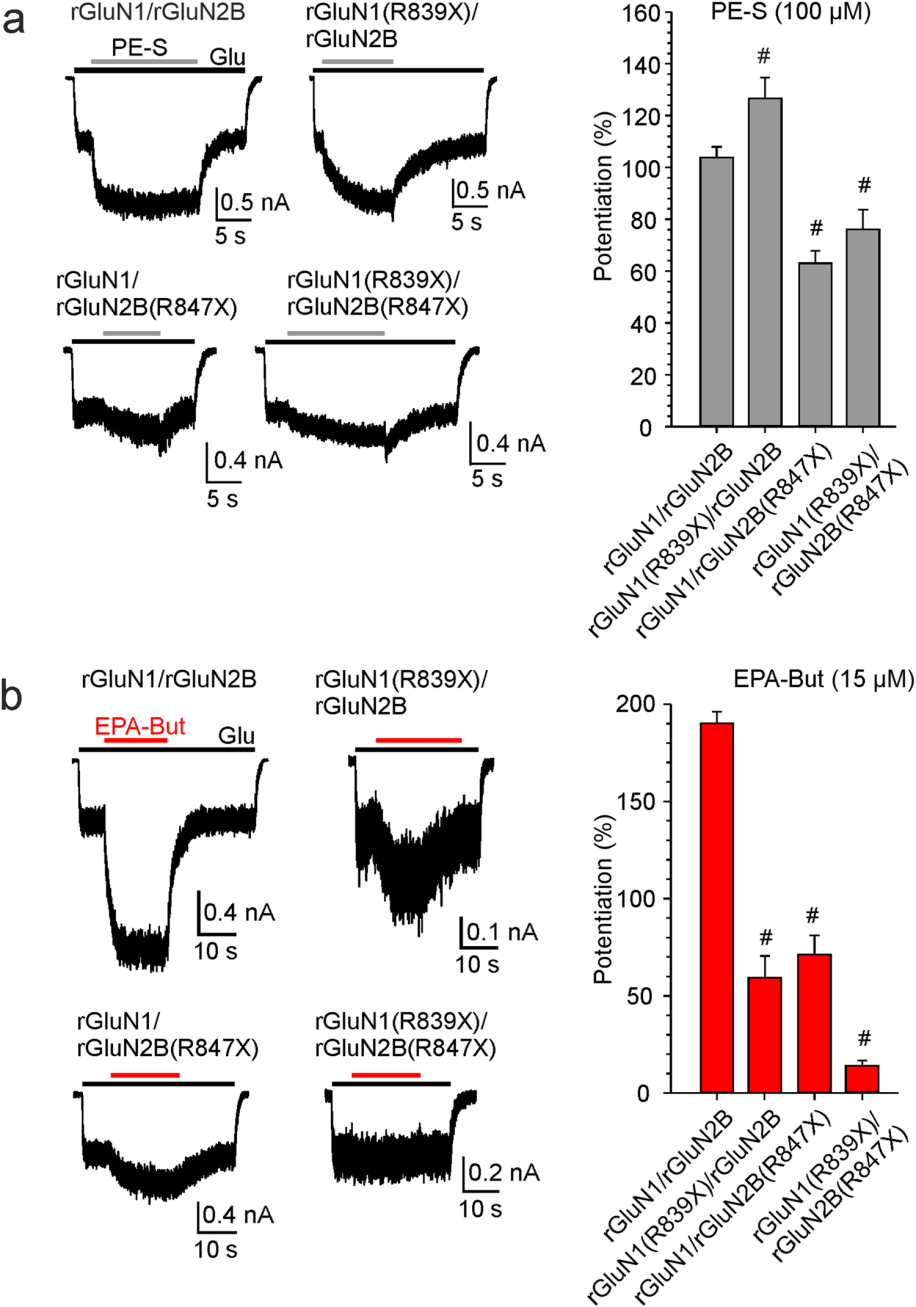
Effects of rGluN1 and rGluN2B CTD truncations on NMDAR sensitivity to steroids. (**a**, **b**) Representative recordings of responses to glutamate (1 µM) before and in the presence of PE-S (100 µM) or EPA-But (15 µM) are shown for HEK cells expressing rGluN1/rGluN2B-WT, rGluN1(R839X)/rGluN2B, rGluN1/rGluN2B(R847X), and rGluN1(R839X)/rGluN2B(R847X) receptors. The bar graphs show the degree of steroid potentiation of glutamate responses induced in rGluN1/rGluN2B receptors with the truncation of the CTD of either rGluN1, rGluN2B, or both subunits. Data represent mean steroid-induced potentiation in % ± SEM (*n* = 13–161). # marks receptors with significantly altered steroid (PE-S/EPA-But) sensitivity (data were power transformed and tested by the ANOVA followed by multiple comparisons versus rGluN1/rGluN2B-WT receptors (Dunnett’s method))

To complete our investigation of how the CTD regions affect NMDAR steroid sensitivity, we compared rat recombinant rGluN1/rGluN2B receptors that lacked the CTD of rGluN1 and/or rGluN2B subunit, containing rGluN1(R839X) and/or rGluN2B(R847X) subunits, respectively. The degree of potentiation induced by PE-S in receptors with most of the rGluN1 CTD truncated (rGluN1(R839X)/rGluN2B) was 136 ± 11% (*n* = 13), which was higher compared to rGluN1/rGluN2B-WT receptors. In contrast, receptors with most of the rGluN2B CTD truncated (rGluN1/rGluN2B(R847X)) were potentiated by PE-S by only 63 ± 5% (*n* = 20), similar to receptors lacking both the rGluN1 and the rGluN2B CTDs (rGluN1(R839X)/rGluN2B(R847X)) (Fig. 8a). In contrast to PE-S, the potentiation induced by EPA-But (15 µM) in rGluN1(R839X)/rGluN2B receptors was reduced to 58 ± 10% (*n* = 13), similar to the result found for rGluN1/rGluN2B(R847X) receptors. Receptors lacking both the rGluN1 and the rGluN2B CTDs were potentiated even less than those with either the rGluN1 or the rGluN2B CTD deleted (Fig. 8b). These data indicate that the rGluN1 CTD deletion affects PE-S and EPA-But potentiation differently, either when present alone or in combination with rGluN2B CTD deletion.

### A region within the C1 cassette of GluN1 regulates the positive allosteric effect of steroids on NMDARs

**Fig. 9:**
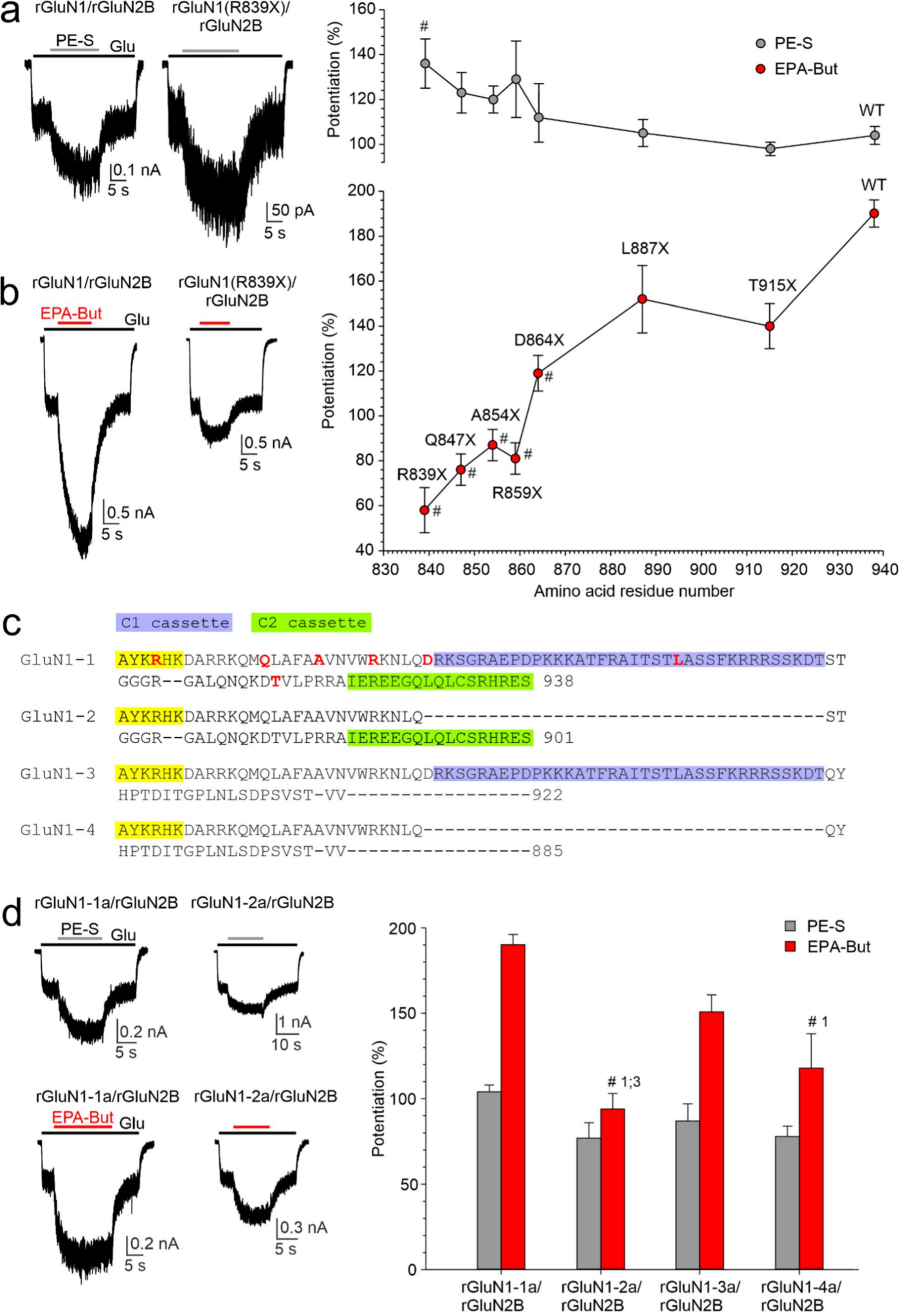
NMDAR steroid sensitivity depends on the rGluN1 splice variant. **a**, **b** Examples of traces obtained from HEK cells expressing rGluN1/rGluN2B-WT and rGluN1(R839X)/rGluN2B receptors. PE-S (100 µM; gray bar) (**a**) and EPA-But (15 µM; red bar) (**b**) were applied in the presence of glutamate (1 µM; black bar). The plot on the right shows the degree of potentiation by PE-S (gray symbols) and EPA-But (red symbols) of responses to glutamate recorded from rGluN1/rGluN2B receptors with the progressively truncated CTD of rGluN1-1a subunit. Data represent mean steroid potentiation in % ± SEM (*n* = 6–13). # marks receptors with significantly altered steroid (PE-S/EPA-But) sensitivity (data were power transformed and tested by the ANOVA followed by multiple comparisons versus rGluN1/rGluN2B-WT receptors (Dunnett’s method)). **c** Amino acid sequence showing a segment of the M4 and the CTD of rGluN1-1a to rGluN1-4a subunits. The membrane region is highlighted in yellow; the sites at which the CTD was truncated are highlighted in red, the C1 cassette is highlighted in blue and the C2 cassette is highlighted in green. **d** Representative recordings of responses to glutamate (1 µM) before and in the presence of PE-S (100 µM) or EPA-But (15 µM) are shown for HEK cells expressing rGluN1-1a/rGluN2B and rGluN1-2a/rGluN2B receptors. The plot on the right shows the relative degree of potentiation by PE-S (gray bars) and EPA-But (red bars) of receptors with different rGluN1 splice variants. Data represent mean steroid-induced potentiation in % ± SEM (*n* = 6–161). Steroid (PE-S/EPA-But) effect data were power transformed and compared using the ANOVA followed by pairwise comparisons (Duncan method). Numbers next to # mark the splice variant which significantly differs

The rGluN1 CTD is comprised of 99 amino acid residues (11% of the total sequence). It contains phosphorylation sites and binding sites for intracellular proteins that regulate membrane trafficking and NMDAR function [4]. To determine the location that controls receptor steroid sensitivity, we analyzed the effect of PE-S and EPA-But on rGluN1/rGluN2B receptors with the rGluN1-1a CTD truncated to different lengths. Figure 9a shows the effect of PE-S (100 µM) on receptors with the stepwise truncated CTD of the rGluN1 subunit. The degree of PE-S-induced potentiation progressively increased with the length of rGluN1 CTD truncation (by 31% for rGluN1(R839X)/rGluN2B receptors in comparison with rGluN1/rGluN2B-WT receptors). In contrast to PE-S, the potentiating effect of EPA-But (15 µM) at receptors containing the rGluN1 subunit with the truncated CTD was strongly reduced when rGluN1(R839X)/rGluN2B and rGluN1/rGluN2B-WT receptors were compared (Fig. 9b).

The region of D864–L887 within the CTD of rGluN1 subunit is critical for the reduction of the EPA-But potentiating effect at rGluN1/rGluN2B receptors. This region comprises amino acid residues that are part of exon 21 (also called the C1 cassette that is present in the GluN1-1a and GluN1-3a splice variants) (Fig. 9c). Therefore, we tested the effect of steroids on rGluN1/rGluN2B receptors containing different splice variants of the rGluN1 subunit. As expected, the degree of potentiation induced by PE-S (100 µM) was similar irrespective of the C1 cassette present (rGluN1-1a to rGluN1-4a)/rGluN2B receptors) (Fig. 9d). The analysis of the effect of EPA-But (15 µM) at rGluN1/rGluN2B receptors containing different splice variants of rGluN1 subunit shows that receptors lacking the C1 cassette were less potentiated by this steroid (Fig. 9d). The analysis of the effect of EPA-But on receptors differing in the presence of the C2 cassette (rGluN1-1a/rGluN2B versus rGluN1-3a/rGluN2B receptors) shows no difference (Fig. 9d).

### The role of the GluN1 CTD in the arrangement of TMD helices

Our recent molecular dynamics (MD) simulations have shown that palmitoylation of juxtamembrane cysteines in the CTD of the GluN2B subunit anchors this CTD to plasma membrane and plays a pivotal role in NMDAR channel opening and closing [10]. Since the GluN1 CTD does not contain cysteine residues that could be palmitoylated [53], we have to consider other mechanisms by which this domain affects EPA-But-induced potentiation. Online analysis (http://crdd.osdd.net/raghava/antibp/) of the whole GluN1-1a CTD amino acid sequence (residues K838–S938) revealed 23 regions with a predicted high propensity for interaction with the membrane. Most of these regions are located partially or completely within the C1 cassette (Supplementary Fig. S2). This offers a hypothesis that the mechanism by which the CTDs from GluN1 and GluN2B subunits affect NMDAR steroid sensitivity may be similar—anchoring the CTD to the plasma membrane and contributing to the subsequent structural changes at the TMD during channel opening and closing.

We have performed metadynamics simulations of the GluN1 CTD embedding into a model membrane using GROMACS with PLUMED. The simulated system consisted of the membrane-bound hGluN1/hGluN2B receptor model with the hGluN1 CTD initially pointing to the water phase. However, the simulations have not identified any stable situation where a significant part of the hGluN1 CTD would be immersed into the membrane. Unbiased MD simulations starting from such positions also quickly led to the hGluN1 CTD being expelled from the membrane and mostly remaining at the membrane surface.

**Fig. 10:**
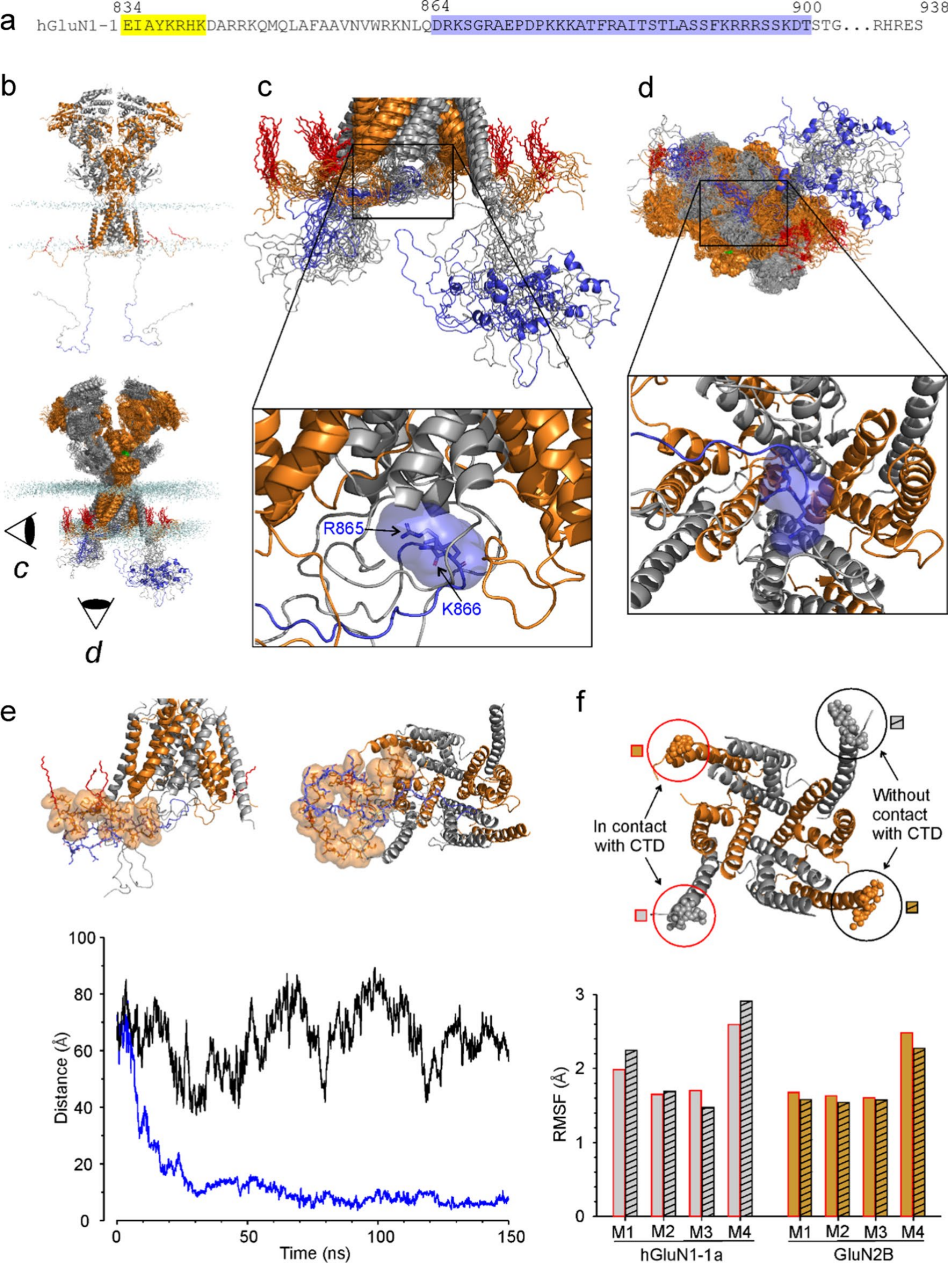
Summary of all-atom MD simulations of the hGluN1/hGluN2B receptor embedded in a model membrane. **a** The amino acid sequence of hGluN1-1a CTD (residues D842 to S938) with the C1 cassette residues (D864 to T900) highlighted in blue. The membrane region is highlighted in yellow. **b**–**f** The hGluN1 subunit is shown as gray cartoon with the C1 cassette (residues D864 to T900) highlighted in blue. The hGluN2B subunit is shown as orange cartoon with the palmitoylated cysteine residues (C849, C854, and C871) highlighted as red sticks. The model includes glycine and glutamate ligands (green molecules) bound in the corresponding ABDs and the receptor is in the open conformation. **b**; *Top* The initial geometry of the hGluN1/hGluN2B receptor used for the MD simulation. Membrane phospholipid P atoms are shown as light blue spheres. The hGluN1 CTDs in the initial model are mostly extended and pointing away from the membrane plane. The palmitoylated fragment of hGluN2B CTD is in contact with the membrane surface, with the palmitoyl tails interacting with the membrane lipids. **b**; *Bottom* Snapshots of hGluN1/hGluN2B receptor geometry from the second half of the MD simulation. Several structures were superimposed. **c**, **d**; *Top* The side view (**c**) and the bottom view (**d**) of the snapshots from the second half of the 100 ns MD simulation show one of the hGluN1 CTDs (gray with blue C1 cassette) near the membrane and the second conformationally more flexible CTD farther from the membrane. **c**, **d**; *Bottom* The positively charged residues R865 and K866 within the C1 cassette of hGluN1 CTD were found in the vicinity of the ion channel exit delineated by M2 helices. **e** The residues in the C1 cassette of hGluN1 CTD also interact with the palmitoylated juxtamembrane region of the hGluN2B CTD. **e**; *Bottom* Plot of the distance between the guanidinium carbon atom of the R865 residue in the C1 cassette of hGluN1 CTD and the center of mass of the *C*_*α*_ atoms of the M2 helix terminal residues L603 (hGluN1) and F600 (hGluN2B). The blue line corresponding to one of the hGluN1 CTDs shows that the R865 cassette residue moves towards the ion channel exit within tens of nanoseconds and stays in its vicinity for the rest of the simulation time. The corresponding residues from the second hGluN1 CTD (black line) remain distant throughout the MD simulation. **f** Summary of TMD helix mobility from the second half of the MD simulation based on positions of the *C*_*α*_ atoms of the M1 residues F583, M2 residues L603, M3 residues F627, and M4 residues K841 of the hGluN1 subunits, and the M1 residues F577, M2 residues F600, M3 residues T626, and M4 residues W844 of the hGluN2B subunits. **f**; *Top* Spheres represent several positions of the M4 terminal residues K841 of hGluN1 (gray) and W844 of hGluN2B (orange) from the second half of the MD simulation. The residues on the left-hand side (in red circles) belong to the TMD side in contact with the hGluN1 CTD residues. The residues in black circles belong to the TMD side without direct contact with the hGluN1 CTD. **f**; *Bottom* The root mean square fluctuations (RMSF) of the above mentioned *C*_*α*_ atoms express the intensity of movement of the TMD helices from the second half of the MD simulation (with the receptor in the open state)

With the possibility of the hGluN1 CTD embedding into the membrane ruled out, we performed additional unbiased all-atom MD simulations of the hGluN1/hGluN2B receptor model containing the full-length hGluN1 subunit (Q05586 residues 23–938) and the hGluN2B subunit with palmitoylated and truncated CTD (Q13224 residues 30 to 877) (Fig. 10b). The results from the representative 150 ns MD simulation are summarized in Fig. 10. The two hGluN1 CTDs behave independently. The positively charged residues R865 and K866 within the C1 cassette of one of the hGluN1 CTDs were found in the vicinity of the ion channel exit, delineated by the M2 membrane helices (Fig. 10c, d). When formed (after about 20 ns of the MD simulation), this interaction is conserved throughout the MD simulation (Fig. 10e), and steric hindrance does not allow the involvement of the corresponding charged residues from the second hGluN1 CTD at the time scale of the MD simulation. At the same time, the hGluN1 CTD C1 cassette also interacts with the palmitoylated juxtamembrane region of the hGluN2B CTD (Fig. 10e). Stabilization of the interacting hGluN1 CTD may translate to a reduced mobility of the hGluN1 transmembrane helices (Fig. 10f). This, together with the anchoring via palmitoylated GluN2B cysteines, could contribute to more efficient activation in response to Gly/Glu ligand binding.

MD simulations of the hGluN1/hGluN2B receptor containing the GluN1-2a GluN1 variant (Q05586-3 residues 23 to 901 corresponding to Q05586 without residues 864 to 900) showed that the hGluN1 CTDs missing the C1 cassette do not significantly interact with the membrane, the ion channel exit residues, or with the hGluN2B palmitoylated juxtamembrane region (Supplementary Fig. S3).

## Discussion

In this study we analyzed ten disease-associated and one artificial nonsense and frame-shift variants resulting in the truncation of the GluN2A or the GluN2B CTD. We have uncovered a range of molecular consequences resulting in altered NMDAR surface expression, synaptic localization, function, and pharmacology.

Our results show that except for GluN2A(Y1387X), all variants exhibited reduced surface expression in HEK/COS-7 cells, and this effect was more profound for receptors containing CTD truncations in the GluN2A than in the GluN2B subunit. All nonsense and frame-shift variants exhibited reduced co-localization with PSD-95 suggesting also their reduced synaptic localization. Surface expression and trafficking of diheteromeric GluN1-1a/GluN2 receptors is primarily controlled by the GluN2 subunit [58, 59]. Endoplasmic reticulum-releasing motif HLFY_839–842_ and HLFY_840–843_ was found within the human GluN2A and GluN2B CTDs, respectively, located directly after the M4 membrane domain [60]. This region is necessary for the assembled receptor complex to exit from the endoplasmic reticulum. The first three amino acids are crucial for the surface expression of functional NMDARs, as they are able to overcome the endoplasmic reticulum retention motifs found within the GluN1 CTD [60–62]. Immediately adjacent to the HLFY motif there are endocytic motifs YWKL_842–845_ and YWQF_843–846_ in the hGluN2A and hGluN2B subunits, respectively, that mediate endocytosis and target internalized receptors along the degradative pathway to late endosomes [63, 64]. Furthermore, a distal endocytic motif YEKL_1474–1477_ discovered in the hGluN2B CTD preferentially promotes the recycling of internalized NMDARs through the early and recycling endosome. In the hGluN2A CTD, a dileucine motif LL_1319–1320_ was found that also regulates receptor internalization [64]. The rate of internalization is further affected by regulatory proteins such as MAGUKs that bind within the CTD of GluN2A and GluN2B subunits and inhibit endocytosis [63–66].

Protein interactions with the CTD are also crucial for receptor trafficking to synapses and for their stabilization at synapses. Most notable is the evolutionarily conserved region ESDV (the last four amino acids within the CTDs of the GluN2A and GluN2B subunits) mediating binding to MAGUK proteins (including PSD-93, PSD-95, SAP97 and SAP102). The GluN2A and GluN2B subunits with missense of truncation variants in the ESDV region reduce the delivery of NMDARs to synapses and impair the anchoring of cell-surface clusters at postsynaptic targets [67–70]. In addition to MAGUKs, numerous other intracellular proteins have been shown to interact with the CTDs and to regulate the levels of synaptic NMDARs. Previous functional analysis of disease-associated missense variants in the GluN2B CTD revealed impaired NMDAR binding to MAGUK proteins (S1415L, L1424F, S1452F), deficiencies in receptor surface expression (S1415L), a decrease in the number of dendritic spines (S1415L), and decreased trafficking and targeting of the receptor to the synapse (S1415L) [71]. Similarly, a missense variant within the GluN2A CTD associated with epilepsy (S1459G) displayed deficits in the NMDAR binding to MAGUK proteins, resulting in trafficking deficits, reduced spine density, and decreased excitatory synaptic transmission [72].

Our data show that for different disease-associated CTD variants, the defects in the NMDAR biogenesis and synaptic localization were combined with altered agonist affinity, increased desensitization, and reduced *P*_o_. These functional changes were confined to the truncations of most of the CTD from GluN2A and GluN2B subunits. Previously, it has been shown that the deletion of the GluN2A or the GluN2B CTD significantly decreased the NMDAR *P*_o_ [7, 73]. Those results were confirmed by single-channel analysis and suggested that the decrease in *P*_o_ is due to longer openings and longer desensitized intervals of NMDARs lacking the GluN2A or the GluN2B CTD. The first 100 amino acids of the GluN2 CTDs were essential for these changes [74]. Recently, we have shown that the NMDAR *P*_o_ was dependent on three palmitoylable cysteines (C849, C854, and C871) located in the juxtamembrane region of the GluN2B CTD [10]. Another effect of the GluN2A CTD on NMDAR properties, namely increased glycine affinity, was also reported [75]. In addition, it was shown that the GluN2A CTD could affect the desensitization of the receptor via calcineurin-mediated dephosphorylation of two serines (S900 and S929) [9].

There is evidence that *GRIN2A* and *GRIN2B* variants are associated with typical phenotypic characteristics: intellectual disability is associated with variants in genes encoding either the GluN2A or the GluN2B subunit, epilepsy is typical for variants in the gene for GluN2A, and autism spectrum disorder is typical for variants in the gene encoding GluN2B [2, 12, 76–78]. However, the molecular mechanisms by which nonsense and frameshift variants in the CTD regions of GluN2A or GluN2B lead to the development of complex neuropsychiatric disorders are not well understood (see Table 1) [79]. One limitation of our results is that the effect of the variants is studied in diheteromeric receptors containing two variant GluN2 copies. We hypothesize that some proportion of the diheteromeric receptors exist in affected heterozygous individuals, but it is likely that most NMDARs are triheteromeric assemblies containing only one variant GluN2 copy. It remains to be determined to what extent one WT GluN2 subunit can rescue the function of the receptor carrying one variant GluN2 copy.

Our study demonstrates that the potentiating effect of steroids PE-S and EPA-But is altered in receptors containing the GluN2A or GluN2B subunits with disease-associated truncations of GluN2A or GluN2B CTDs. Steroid effects are reduced or inverted to inhibition for receptors with an almost complete deletion of the GluN2 CTD. We have explored the molecular mechanisms of steroid effects in detail and found that stepwise truncation of the GluN2B CTD affected the potentiating effect of EPA-But and PE-S similarly and that the critical region involved a narrow stretch of amino acid residues close to the membrane (Fig. 5). Subsequently, we have identified three palmitoylable cysteine residues (C849, C854, and C871) [53], to be critically involved in the control of the steroid-induced potentiation of NMDARs. We have shown recently that the palmitoylation of these cysteines also affects the NMDAR sensitivity to inhibitory steroids [10]. This suggests that juxtamembrane palmitoylation of NMDARs reciprocally controls the action of inhibitory steroids at the extracellular channel vestibule and the effect of potentiating steroids at the TMD-lipid interface [80, 81].

Additional experiments revealed that steroid-induced potentiation is affected by GluN1 and GluN2 CTD truncation in a steroid-specific and subunit-specific manner. Consistent with previous data, PE-S had a similar or stronger potentiating effect on rGluN1(R839X)-containing receptors compared to rGluN1/rGluN2B-WT receptors but showed reduced potentiation of receptors containing rGluN2B(R847X) subunit [82]. The potentiating effect of a novel synthetic steroid EPA-But [27] was reduced at receptors containing rGluN1(R839X) and it was even more decreased in receptors with both the rGluN1 and the rGluN2B CTD deleted (Fig. 8b). A stepwise truncation of the rGluN1 CTD indicated that the potentiating effect of EPA-But was dependent on the juxtamembrane region (R839 to L887) of rGluN1 CTD, while only the complete rGluN1 CTD truncation affected potentiation by PE-S (Fig. 9a).

We have shown that EPA-But, a steroid with a “bent” structure, shares a disuse-dependent mechanism of a potentiating effect at NMDARs with “planar” steroids like PE-S [83]. However, the sites of action for EPA-But and PE-S at the NMDAR are different [27]. Alanine scan mutagenesis in combination with in-silico analysis indicated that EPA-But binds to the TMD domain of the NMDAR at three distinct sites localized at the interfaces of TMD helices (GluN1(M4)/GluN2B(M1), GluN2B(M1/M4), and GluN2B(M4)/GluN1(M1) interfaces). The site between GluN2B(M1/M4) helices is shared with PE-S, but the residues important for the binding of the two steroids do not overlap [27, 80]. During the channel transition from the closed to the open state, the ATD, ABD, and the extracellular segments of the M3 helices of the GluN1 and GluN2B subunits rotate around the longitudinal axis of the receptor [31, 84]. Reduced mobility of the external layer of the TMD helices in response to the additional anchoring of the GluN1 CTD to the membrane could contribute to a more productive transfer of the mechanical signal during the opening transition of the receptor. Further, the EPA-But binding to the site identified at the interface of the GluN1(M4)/GluN2B(M1) helices [27] could be more efficient when fluctuations of the surrounding helices are lower.

We show that the C1 cassette in the GluN1 subunit CTD plays a key role in the GluN1-mediated control of NMDAR sensitivity to EPA-But. As with other glutamate receptor subunit genes, different GluN1 subunit splice variants are expressed in the central nervous system with developmental and regional differences [58, 85–87]. The GluN1-2 splice variant (lacking the C1 cassette), showing reduced potentiation by EPA-But, is predominantly expressed in the neonatal rat brain. In contrast, in the adult brain, it is co-expressed together with the GluN1-1 and GluN1-4 splice variants. While the GluN1-2 splice variant is homogeneously expressed, GluN1-1 and GluN1-4 splice variants show complementary distributions, with the GluN1-1 splice variant concentrated in rostra1 structures (e.g., olfactory bulb, cortex, caudate, hippocampus) [58]. Therefore, the modulatory effect of EPA-But at NMDARs will likely be affected by the spatial and temporal patterns of GluN1 subunit splice variant expression.

In conclusion, NMDA receptors carrying disease-associated nonsense or frameshift variants resulting in the truncation of the CTD of the GluN2A or the GluN2B subunit are hypofunctional and characterized by reduced surface expression and synaptic localization. Furthermore, the activity of variant NMDARs is modulated by neuroactive steroids in a manner that is steroid-specific, subunit-specific, and GluN1 splice variant-specific, demonstrating the potential for developing new therapeutic neurosteroid-based ligands to treat diseases associated with the hypofunction of the glutamatergic system.

### Supplementary Information

Below is the link to the electronic supplementary material.

Supplementary file1 (DOCX 1067 kb)

## Data Availability

The datasets generated during and/or analyzed during the current study are available from the corresponding author on reasonable request.

## Abbreviations

ABD: Agonist-binding domain
ANOVA: Analysis of variance
ATD: Amino-terminal domain
CTD: C-terminal domain
DAPI: 4′,6-Diamidino-2-phenylindole
DIV: Day in vitro
EDTA: Ethylenediaminetetraacetic acid
EPA-But: 4-(20-Oxo-5β-pregnan-3β-yl) 3-butanoic acid, epipregnanolone butanoic acid
HEK: Human embryonic kidney
hGluN1: Human GluN1-1a subunit (unless otherwise stated)
MD: Molecular dynamics
MK801: (5S,10R)-(+)-5-methyl-10,11-dihydro-5H-dibenzo[a,d]cyclohepten-5,10-imine hydrogen maleate
NMDA: *N*-methyl-d-aspartate
NMDAR: *N*-methyl-d-aspartate receptor
*P*_o_: Channel open probability
PE-S: 20-Oxo-pregn-5-en-3β-yl 3-sulfate, pregnenolone sulfate
rGluN1: Rat GluN1-1a subunit (unless otherwise stated)
RMSF: Root mean square fluctuations
SEM: Standard error of the mean
TMD: Transmembrane domain
WT: Wild type
