# Supplementary material for: Disease-associated nonsense and frame-shift variants resulting in the truncation of the GluN2A or GluN2B C-terminal domain decrease NMDAR surface expression and reduce potentiating effects of neurosteroids
Source: Cell Mol Life Sci. 2024 Jan 12;81(1):36. doi: 10.1007/s00018-023-05062-6 (PMC10786987; doi:10.1007/s00018-023-05062-6)
Supplement: Supplementary file 1 — Supplementary file1 (DOCX 1067 kb) [file 18_2023_5062_MOESM1_ESM.docx]

# Supplementary Information

Disease-associated nonsense and frame-shift variants resulting in the truncation of the GluN2A or GluN2B C-terminal domain decrease NMDAR surface expression and reduce potentiating effects of neurosteroids

Bohdan Kysilov^#1^, Viktor Kuchtiak^#1,4^, Barbora Hrcka Krausova^1^, Ales Balik^1^, Miloslav Korinek^1^, Klevinda Fili^1,5^, Mark Dobrovolski^1,5^, Vera Abramova^1,5^, Hana Chodounska^2^, Eva Kudova^2^, Paulina Bozikova^3^, Jiri Cerny^1^, Tereza Smejkalova^1^, and Ladislav Vyklicky^1^

**Supplementary Fig. S1** Summary of glutamate-evoked current density in cells expressing NMDA receptors with truncated GluN2 subunits.

**Supplementary Fig. S2** Prediction of membrane interaction sites in the GluN1-1 CTD.

**Supplementary Fig. S3** Summary of all-atom MD simulations of the hGluN1/hGluN2B receptor containing the Glun1-2a GluN1 variant (Q05586-3 residues 23 to 901 corresponding to Q05586 without residues 864 to 900) and the truncated palmitoylated GluN2B CTD (Q13224 residues 30 to 877) embedded in a model membrane.


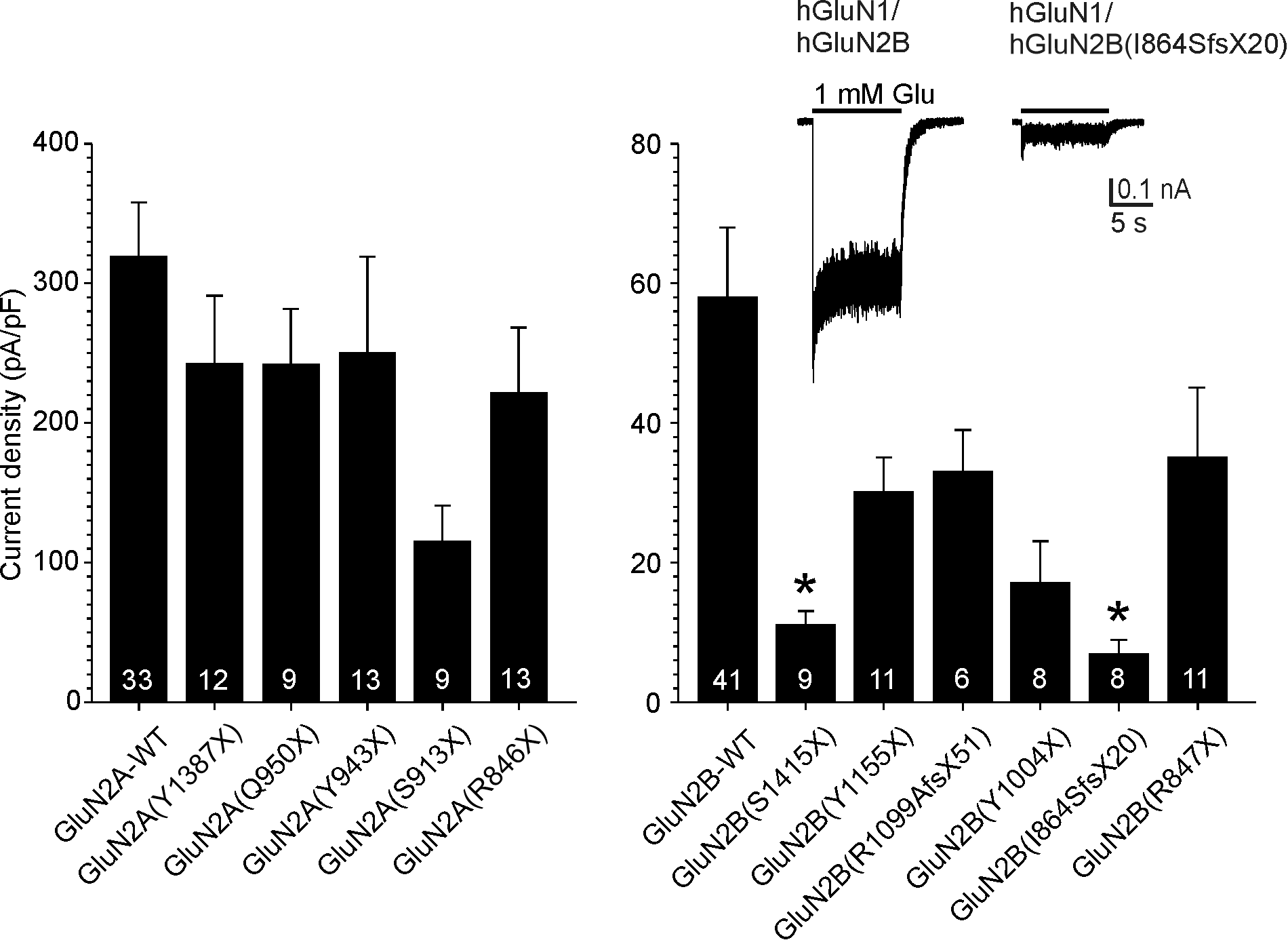


##### Figure S1

**Supplementary Figure S1** **|** **Summary of glutamate-evoked current density in cells expressing NMDA receptors with truncated GluN2 subunits.** Graphs show the summary of mean current amplitudes ± SEM (*n*) evoked by 1 mM glutamate and normalized with respect to the individual HEK cell capacitance. GluN2A: one-way ANOVA *p* = 0.137; GluN2B: one-way ANOVA *p* = 0.015 followed by multiple comparisons *versus* GluN1-1a/GluN2B-WT (Holm-Sidak method): *p* = 0.029 (S1415X), *p* = 0.031 (I864SfsX20). Inset shows representative whole-cell recordings of currents induced in HEK cells transfected with genes encoding hGluN1-1a/hGluN2B-WT and hGluN1˗1a/hGluN2B(I864SfsX20). Glutamate (1 mM, black bar) was applied for 10 s. Cells were bathed in glycine (30 µM).


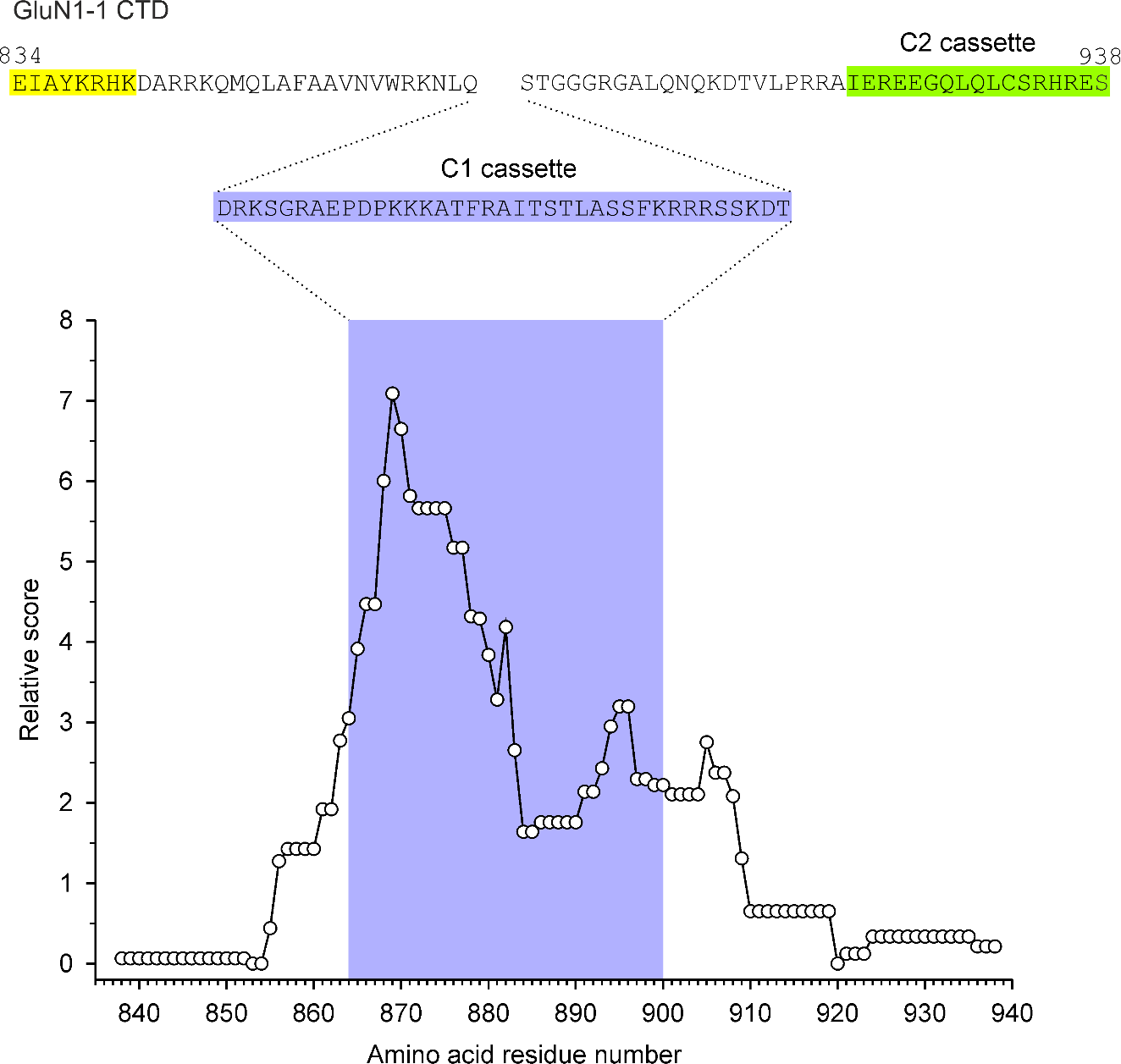


##### Figure S2

**Supplementary Figure S2 | Prediction of membrane interaction sites in the GluN1-1 CTD.** The whole CTD of GluN1-1 protein (E834–S938) was subjected to the prediction of propensity for interaction with the membrane using AntiBP Server (<http://crdd.osdd.net/raghava/antibp/>). The prediction was done by using Support Vector Machine-based method [1]. The relative score for each amino acid residue in the CTD (K838 to S920) is a sum of scores computed for 23 regions with a predicted high propensity for interaction with the membrane. Cytoplasmic portion of the M4 helix is highlighted in yellow; C1 cassette is highlighted in blue; C2 cassette is highlighted in green*.*

1. Lata S, Sharma BK, Raghava GP (2007) Analysis and prediction of antibacterial peptides. BMC Bioinformatics 8:263. <https://doi.org/10.1186/1471-2105-8-263>


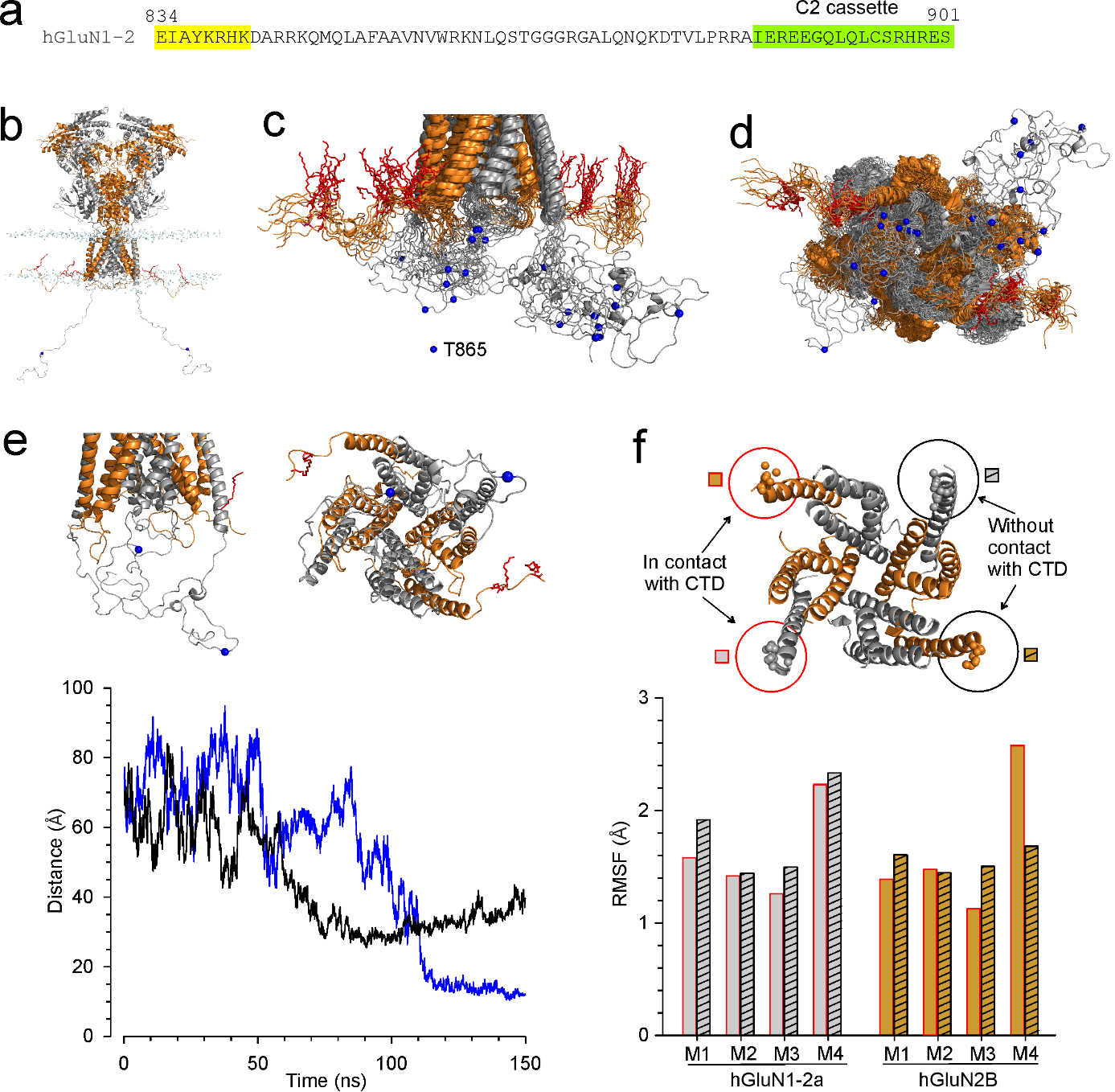


##### Figure S3

**Supplementary Figure S3** **|** **Summary of all-atom MD simulations of the hGluN1/hGluN2B receptor containing the Glun1-2a GluN1 variant (Q05586-3 residues 23 to 901 corresponding to Q05586 without residues 864 to 900) and the truncated palmitoylated GluN2B CTD (Q13224 residues 30 to 877) embedded in a model membrane.** (**a**) The GluN1-2 (Q05586-3) CTD sequence (residues 834 to 901) with the C2 cassette residues 885 to 901 highlighted in green. The membrane region is highlighted in yellow. (**b-f**) The GluN1 subunit is shown as a gray cartoon with the T865 residue (corresponding to the R865 C1 cassette residue of the canonical Q05586 variant) highlighted in blue. The GluN2B is shown as an orange cartoon with the palmitoylated Cys residues (C849, C854, and C871) highlighted as red sticks. The model includes glycine and glutamate ligands in the corresponding ABDs and the receptor is in the open conformation. (**b**) The initial geometry of the GluN1-2a/GluN2B receptor used for the MD simulation. Membrane phospholipid P atoms are shown as light blue spheres. The GluN1 CTDs in the initial model are mostly extended and pointing away from the membrane plane. The GluN2B CTD palmitoylated fragment is in contact with the membrane surface with the palmitoyl tails interacting with the membrane lipids. The side view (**c**) and the bottom view (**d**) at several superimposed snapshots from the second half of the 100 ns MD simulation showing that both GluN1 CTDs are conformationally flexible and do not interact significantly with the channel exit residues. (**e**; *Top*) The CTD of the GluN1-2a splice variant also does not significantly interact with the palmitoylated juxtamembrane region of the GluN2B CTD (contrary to the canonical GluN1-1a variant). (**e**; *Bottom*) Plot of the distance between the C_β_ atom of the GluN1 CTD residue T865 (corresponding to the R865 C1 cassette residue of the canonical Q05586 variant in **Fig. 10e**) and the center of mass of the C_α_ atoms of the M2 helix terminal residues L603 (GluN1) and F600 (GluN2B). The blue line corresponding to one of the GluN1 CTDs shows that the T865 residue moves towards the ion channel exit after about 110 nanoseconds of the simulation but the distance is still significantly larger than that of the canonical GluN1 R865 and no direct interactions can be detected. The corresponding residues from the second GluN1 CTD (black line) remain distant throughout the simulation. (**f**) Summary of TMD helix mobility from the second half of the MD simulation based on positions of the C_α_ atoms of the M1 residues F583, M2 residues L603, M3 residues F627, and M4 residues K841 of the GluN1 subunit, and the M1 residues F577, M2 residues F600, M3 residues T626, and M4 residues W844 of the GluN2B subunit. (**f**; *Top*) Spheres represent several positions of the M4 terminal residues K841 of the GluN1 (gray) and W844 of the GluN2B (orange) from the second half of the MD simulation. The positions on the left hand side (in red circles) correspond to the TMD side with the GluN1 CTD residues closer to the membrane. The positions in black circles correspond to the conformationally more flexible GluN1 CTD. (**f**; *Bottom*) The root mean square fluctuations (RMSF) of the TMD helices from the second half of the MD simulation (with the receptor in the open state). The plot shows that the M4 terminal residues are more mobile compared to the rest of the transmembrane helices, but there are no significant differences between the two GluN1 subunits. The mobility of the GluN2B helices is also comparable and on the scale of the GluN2B fluctuations observed for the side of the canonical GluN1-1/GluN2B without contact with the C1 cassette residues.
